# Overcoming the Tumor Collagen Barriers: A Multistage Drug Delivery Strategy for DDR1‐Mediated Resistant Colorectal Cancer Therapy

**DOI:** 10.1002/advs.202402107

**Published:** 2024-07-02

**Authors:** Guangman Cui, Shaohui Deng, Biao Zhang, Manchun Wang, Zhousheng Lin, Xinyue Lan, Zelong Li, Guangyu Yao, Meng Yu, Jun Yan

**Affiliations:** ^1^ Department of General Surgery & Guangdong Provincial Key Laboratory of Precision Medicine for Gastrointestinal Tumor Nanfang Hospital Southern Medical University Guangzhou 510515 China; ^2^ The Tenth Affiliated Hospital of Southern Medical University Dongguan Guangdong 523059 China; ^3^ NMPA Key Laboratory for Research and Evaluation of Drug Metabolism & Guangdong Provincial Key Laboratory of New Drug Screening & Guangdong‐Hongkong‐Macao Joint Laboratory for New Drug Screening School of Pharmaceutical Sciences Southern Medical University Guangzhou 510515 China; ^4^ Breast Center Department of General Surgery Nanfang Hospital Southern Medical University Guangzhou 510515 China; ^5^ Zhujiang Hospital, Southern Medical University Guangzhou 510282 China; ^6^ Department of Gastrointestinal Surgery Shenzhen People's Hospital Second Clinical Medical College of Jinan University First Affiliated Hospital of Southern University of Science and Technology Shenzhen Guangdong 518020 China

**Keywords:** drug delivery, ecm modulation, multistage nanomedicine, sirna nanocarriers

## Abstract

The extracellular matrix (ECM) is critical for drug resistance in colorectal cancer (CRC). The abundant collagen within the ECM significantly influences tumor progression and matrix‐mediated drug resistance (MMDR) by binding to discoidin domain receptor 1 (DDR1), but the specific mechanisms by which tumor cells modulate ECM via DDR1 and ultimately regulate TME remain poorly understand. Furthermore, overcoming drug resistance by modulating the tumor ECM remains a challenge in CRC treatment. In this study, a novel mechanism is elucidated by which DDR1 mediates the interactions between tumor cells and collagen, enhances collagen barriers, inhibits immune infiltration, promotes drug efflux, and leads to MMDR in CRC. To address this issue, a multistage drug delivery system carrying DDR1‐siRNA and chemotherapeutic agents is employed to disrupt collagen barriers by silencing DDR1 in tumor, enhancing chemotherapy drugs diffusion and facilitating immune infiltration. These findings not only revealed a novel role for collagen‐rich matrix mediated by DDR1 in tumor resistance, but also introduced a promising CRC treatment strategy.

## Graphical abstract

A multistage nanomedicine (si/SN38‐NP) actively targets to tumor ECM modulation, where si/SN38‐NP are activated to liberate si‐NP and SN38‐NP. si‐NP penetrates into tumor cells quickly due to its small size thereby silencing DDR1 and regulating collagen barriers. Tumor permeability is thus increased to enhance SN38‐NP penetration and immune cells tumoral infiltration, finally achieving a synergistic chemo‐immunotherapy effect in colorectal cancer.

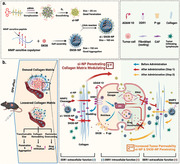

## 1 Introduction

Biological barriers hinder drug delivery and limit their effectiveness at tumor sites.^[^ ^1^ ^]^ Cancer associated fibroblasts (CAFs) in tumor microenvironment (TME) produce and deposit abundant collagen around tumor cells, altering the structure and stiffness of extracellular matrix (ECM). The dense collagen matrix constructs biological barriers that hinder the access of exogenous drugs, antibodies and immune cells to the tumor site.^[^ ^2^ ^]^ Additionally, CAFs‐induced collagen deposition and remodeling can enhance the interaction between tumor cells and collagen, providing a signaling platform that promoting tumor cell survival, leading to drug resistance.^[^ ^3^ ^]^

Drug resistance is still a dilemma in the treatment of colorectal cancer (CRC). Currently, most anti‐cancer therapies merely target to cancer cells, largely ignoring the influence of tumor stroma. However, the tumor matrix promotes cancer cell survival and recurrence, resulting in fatal disease.^[^ ^4^ ^]^ Therefore, combining anticancer cell drugs with matrix modulating strategy to overcome drug resistance and improve CRC survival outcomes is attractive but challenging.

Several preclinical studies have focused on ECM modulation by targeting ECM molecules, ECM remodeling enzymes or regulating fibroblast function.^[^ ^5^ ^]^ But current researches and clinical trial results showed that existing methods of targeting the matrix are not very efficient. For example, the phase II trial using Talabostat to inhibit Fibroblast activation protein‐α (FAP)‐positive fibroblasts to reduce stroma production failed to demonstrate clinical efficacy in CRC.^[^ ^6^ ^]^ The phase III trial on PEG‐hyaluronidase only increased the objective response rate (O but failed to improve overall survival (OS) or progression‐free survival (PFS) in hyaluronan‐high metastatic pancreatic adenocarcinoma.^[^ ^7^ ^]^ Among lots of cancer treatment options targeting ECM, one of the most successful cases is the application of tyrosine kinase inhibitors (TKIs) in non‐small cell lung cancer with EGFR mutation and chronic myeloid leukemia, which is partially mediated by the blockage of the signal transduction stimulated by discoidin domain receptors (DDRs).^[^ ^8^ ^]^ DDRs may be potential targets, but few studies have focused on the regulation of ECM by DDRs. In addition, the structural complexity of ECM and intratumoral heterogeneity are not fully understood, which may limit the application of targeted therapies for ECM. Developing new therapies to modulate tumor stroma requires better understanding of the interaction between tumor cells and the collagen matrix, particularly, how cancer cells transmit signals leading to the formation of tumor‐protective TME.

The abundant collagen significantly influences tumor progression and drug resistance by binding to tumor cell surface receptors. DDR1, a collagen receptor, is the tyrosine kinase receptors subfamily widely expressed on the surface of tumor cells.^[^ ^9^ ^]^ DDR1 overexpression promotes the tumor‐collagen interactions in ECM, leading to fibroblasts activation and increased collagen production.^[^ ^10^ ^]^ Phosphorylation of DDR1 and DDR2 on linear collagen fibers has been reported to associated with matrix‐mediated drug resistance (MMDR) in melanoma.^[^ ^11^ ^]^ DDR1 has become a new target for tumor therapy as its role in regulating MMDR. However, the functional role of DDR1 activity in mediating tumor resistance in CRC has been poorly documented. It is currently unclear how cancer cell receptors affect the collagen matrix, especially how blocking receptors can modulate ECM.

As drug carriers, nanomaterials have greatly improved drug delivery efficiency, and improved drug accumulation within tumors through enhanced permeability and retention (EPR) effects.^[^ ^12^ ^]^ However, the actual performance of these nanomedicine in vivo and in clinic is still not satisfying. Addressing the issue of difficulty for nanomedicine to penetrate dense collagen matrix and penetrate deep into tumors, we designed a multistage responsive nanomedicine co‐delivering DDR1‐siRNA and chemotherapeutic drug SN38 (si/SN38‐NP). Irinotecan (CPT‐11) is a first‐line chemotherapy for advanced or metastatic colorectal cancers. SN‐38 is the active metabolite of CPT‐11 and its anti‐tumor ability is 100 times that of CPT‐11. However, SN‐38 has poor water solubility, low bioavailability and severe dose‐limiting toxicity so it has not been successfully used in clinic.^[^ ^13^ ^]^ The co‐delivery of SN38 by a nanoparticle drug carrier system can overcome the above limitations and exert the highly effective anti‐tumor activity of SN38.^[^ ^14^ ^]^

The designed si/SN38‐NP has strong penetration ability and enhanced tumor aggregation due to its tunable sizes, which releases small‐size siRNA particles and larger‐size SN‐38 particles in response to the highly expressed matrix metalloproteinases (MMPs) in the TME. Thereby the siRNA particles penetrate into tumor cells quickly due to its small size to modulate collagen barriers through silencing DDR1. Then the tumor permeability was thus increased, enhancing the tumor penetration of larger‐size SN‐38 particles and immune cells infiltration, blocking cancer cell signaling, thereby overcoming CRC chemotherapy resistance. In this study, we innovatively validated the specific mechanism of DDR1 in mediating MMDR in CRC and proposed an effective strategy to enhance the antitumor efficacy by actively modulating the tumor collagen matrix during chemotherapy.

## 2 Results

### 2.1 Association of the Overexpression of DDR1 with Collagen Score in CRC Chemotherapy Resistance

**Figure 1:**
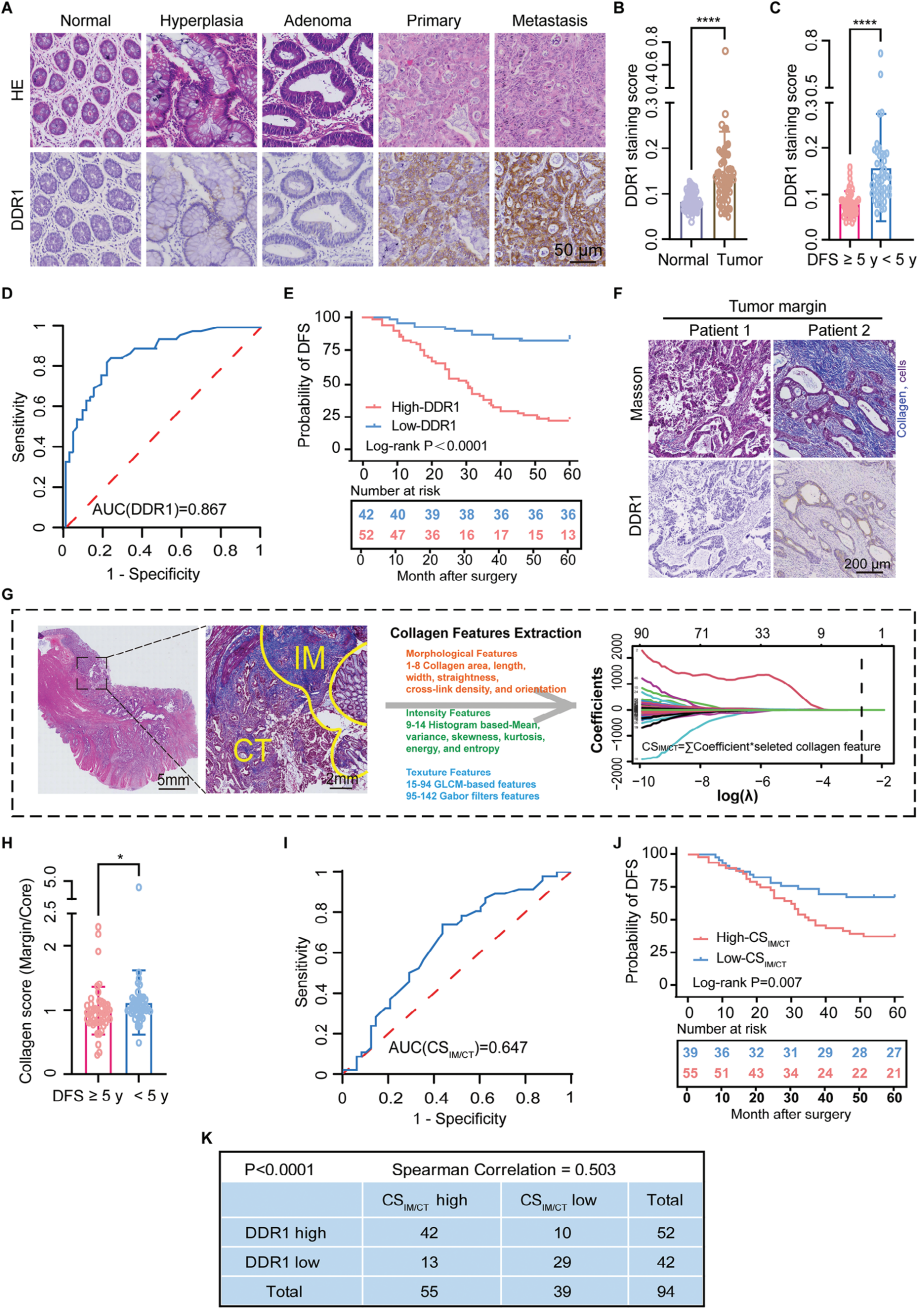
Association of the overexpression of DDR1 with collagen signature in CRC chemotherapy resistance. A) Representative IHC images of DDR1 expression and H&E staining in human tissues from normal colorectum, hyperplasia, adenoma, primary colorectal carcinoma and the liver metastases. Scale bar, 50 µm. B) The comparative analysis of DDR1 histological scoring between normal and tumor tissues (*n* = 60 per group). Samples were scored for DDR1 expression by Image J. Mann‐Whitney Test, two tailed. *P < 0.05, **P < 0.01, ***P < 0.001, ****P < 0.0001. C) The comparative analysis of DDR1 histological scoring between patients with DFS≥5 years and DFS<5 years (*n* = 48 vs *n* = 46). The samples were performed in a blinded fashion. Mann‐Whitney Test, two tailed. *P < 0.05, **P < 0.01, ***P < 0.001, ****P < 0.0001. D) Time‐dependent ROC curve of the DDR1 staining score for predicting 5‐year DFS in all patients. E) Relationship of the DDR1 staining score with disease‐free survival in the cohort. F) Representative images of Masson staining colorectal tumor tissues with low (Patient 1) and high (Patient 2) DDR1 expression, respectively. Scale bar, 200 µm. G) Flowchart of CS_IM/CT_ construction. A total of 142 collagen features were extracted from Masson staining images in both core of the tumor (CT) and invasive margin (IM) for each sample. Predictive collagen features were selected using LASSO regression in the cohort, and the collagen score was constructed using the calculation formula. The integrated collagen score of dividing IM by the CT (CS_IM/CT_) was established and further analyzed. H) The comparative analysis of CS_IM/CT_ between patients with DFS≥5 years and DFS<5 years. Mann‐Whitney Test, two tailed. *P < 0.05, **P < 0.01, ***P < 0.001, ****P < 0.0001. I) Time‐dependent ROC curve of the CS_IM/CT_ for predicting 5‐year DFS in all patients. J) Relationship of the CS_IM/CT_ with disease‐free survival in the cohort. K) Correlation between CS_IM/CT_ and DDR1 staining score in above specimens. Rank Correlation, two‐tailed.

Collagen alterations in the TME may provide valuable information about prognosis and response to chemotherapy.^[^ ^15^ ^]^ Previous studies have demonstrated the critical role of DDR1 in collagen arrangement.^[^ ^16^ ^]^ However, the detailed alterations of high‐dimensional quantitative collagen features associated with DDR1 regulation, including morphologic and textural features remain unclear. First, to validate the correlation between DDR1 and MMDR in clinical cases, we compared the expression of DDR1 in normal colorectal tissue, adenomas, malignant primary CRC lesions and metastatic lesions. The expression levels of DDR1 significantly increased in tumors compared to normal tissues during tumor progression, indicating that DDR1 is a promising prognostic factor for CRC (**Figure** **1**A,B). 94 patients were retrospectively analyzed and divided into two groups according to 5‐year disease free survival (DFS) (demographic characteristics of patients were summarized in Table S1, Supporting Information). Notably, the expression levels of DDR1 were significantly higher in patients with DFS<5 years compare to those with DFS≥5 years (Figure 1C). Time‐dependent receiver operating characteristic (ROC) analysis exhibited good discrimination of 5‐year DFS with an AUC of 0.867 (Figure 1D). Kaplan‐Meier survival analysis further demonstrated significantly longer estimated mean 5‐year DFS in the low‐DDR1 group compared to the high‐DDR1 group (54.2 months vs 33.8 months, *p* < 0.0001) (Figure 1E). Masson staining images revealed denser and more aligned collagen fibers, especially at the tumor margin, in patients with high DDR1 expression (Figure 1F). This prompted detailed collagen features analysis and further quantification of the collagen barriers. High‐dimensional features could be extracted through image analysis and algorithm techniques to describe the quantitative alterations of collagen. Our previous research has shown that integrating multiple features into a single index to construct a collagen score (CS) for evaluating tumor prognosis will help clinical decision making.^[^ ^17^ ^]^

To better understand the collagen distribution and configuration, 142 collagen features (the list of collagen features are presented in Table S2, Supporting Information) were extracted from invasive margin (IM) and core of tumor (CT) regions of each patient's Masson staining images, respectively. Using least absolute shrinkage and selection operator (LASSO) regression, 2 potential features were selected to construct a CS from 142 collagen features in the cohort. The CS was positively correlated with collagen entropy and GLCM‐based^84th^ features. Entropy is one of the collagen intensity parameters. The GLCM‐based^84th^ features belong to the texture parameter which describe the spatial distribution of collagen. Texture parameter can be used to describe the arrangement of the components of any substance. This result indicates that the differences in collagen intensity at spatial distribution between IM and CT may be the key factor driving CRC MMDR. Hence, we calculated the interregional variation CS_IM/CT_ as an indicator to quantify tumor collagen remodeling (Figure 1G). A significant increase of CS_IM/CT_ was revealed in patients with DFS <5 years compared to those with DFS≥5 years (Figure 1H). ROC analysis demonstrated good discrimination for 5‐year DFS with an AUC of 0.647 (Figure 1I). Kaplan‐Meier survival analysis indicated that the low‐CS_IM/CT_ group had significantly longer estimated mean 5‐year DFS compare to the high‐CS_IM/CT_ group (48.8 months vs 38.7 months, *p* = 0.007) (Figure 1J). These results suggest that the CS_IM/CT_ is an indicator in detecting the risk for CRC MMDR. Moreover, a strong positive correlation (Spearman Correlation = 0.503) was observed between DDR1 staining score and CS_IM/CT_ (Figure 1K), supporting the proposition that elevated DDR1 expression drives collagen remodeling to instigate MMDR.

### 2.2 DDR1‐Dependent Collagen Remodeling Hinders the Infiltration of Anti‐Tumor Immune Cells

Abnormal collagen deposition around tumors is mainly produced by CAFs. In order to better simulate the supporting function of CAFs in the TME, we activated fibroblasts into CAFs (Figure S1A, Supporting Information),^[^ ^18^ ^]^ and examined the expression of α‐smooth muscle actin (α‐SMA) and FAP (Figure S1B–C, Supporting Information). One step further, we established a CAFs‐mediated resistant mice model through subcutaneous co‐injecting CAFs and MC38 cells (Figure S1D, Supporting Information).^[^ ^19^ ^]^ Co‐injection significantly promoted tumor growth in mice (Figure S1E–G, Supporting Information). Masson staining showed that there was more abundant collagen in the tumor of co‐injection group, with higher collagen fiber length and cross‐linking space (Figure S1H–J, Supporting Information). In addition, elicited significant changes in tumor elasticity were found. Higher stress relaxation and Young's modulus values, the mechanical parameters reflecting stiffness of a solid material, were found in the co‐injection group (Figure S1K,L, Supporting Information). Therefore, the CAFs‐mediated resistant mice model has higher stiffness and modulated tumor biomechanics of the tumor.

**Figure 2:**
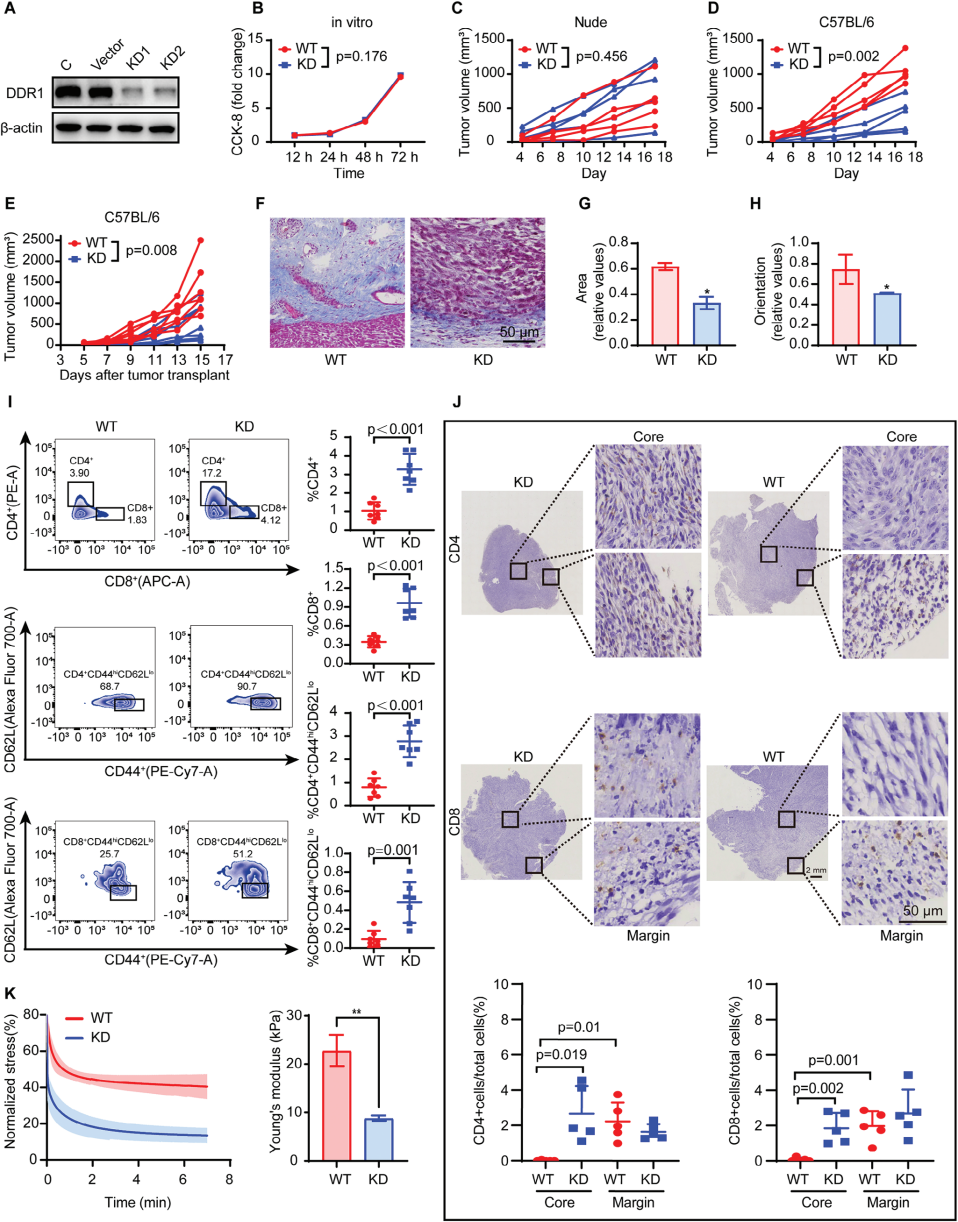
DDR1‐dependent ECM remodeling hinders infiltration of anti‐tumor immune cells. A) Immunoblotting analysis of DDR1 in MC38 cells. B) In vitro cell proliferation of MC38 cells. *n* = 3. Two‐way ANOVA, two tailed. C,D) MC38 tumor growth curves of immunodeficient nude mice model (*n* = 5) (C) and immunocompetent C57BL/6 mice (*n* = 5) (D). MANOVA Of Repeated Measuring, two tailed. E) MC38 tumor growth curves of mice model, where MC38 tumors were grown on immunodeficient hosts, following by cutting the tumors into ≈60 mg pieces and transplanted into C57BL/6 mice (*n* = 7). MANOVA Of Repeated Measuring, two tailed. F) Representative images of Masson stanning of tumors. Scale bar, 50 µm. G,H) The collagen features (area and fiber orientation) extracted from Masson staining images were quantitatively analyzed by MATLAB software. *n* = 3 tumors per group. Student's t‐test, two tailed. *P < 0.05, **P < 0.01, ***P < 0.001, ****P < 0.0001. I) Flow cytometry analysis of tumor‐infiltrating total CD8^+^ and CD4^+^ T cells and activated (CD44^hi^CD62L^lo^) CD8^+^ and CD4^+^ T cells in MC38 Ddr1‐WT/KD tumor. *n* = 5. Student's t‐test, two tailed. J) Representative images of CD4^+^ and CD8^+^ T cell staining at MC 38 Ddr1‐WT/KD tumor margins and tumor cores. Quantification of CD4^+^ T cell and CD8^+^ T cell IHC at MC38 Ddr1‐WT/KD tumor margin and core. *n* = 5. Student's t‐test, two tailed. K) Stress relaxation of tumors in the two group. Shaded regions are s.d. of normalized stresses of different samples. Young's modulus of as‐treated tumors. *n* = 3 tumors per group. Student's t‐test, two tailed. *P < 0.05, **P < 0.01, ***P < 0.001, ****P < 0.0001.

Then, we constructed a stable Ddr1‐KD MC38 cell line (**Figure** **2**A), showing no influence on tumor cell proliferation in vitro (Figure 2B). Nevertheless, Ddr1‐KD did not impact tumor growth in CAFs co‐injecting nude mice (Figure 2C; Figure S2A, Supporting Information), whereas it inhibited tumor growth in CAFs co‐injecting immunocompetent C57 mice (Figure 2D; Figure S2B, Supporting Information). These findings inspired us to address the question of how DDR1 expression probably influenced the tumor immune microenvironment.

Further experiments involved tumor transplantation from immunodeficient nude mice to immunocompetent C57 mice, resulting in significant tumor growth inhibition in the Ddr1‐KD group (Figure 2E; Figure S2C, Supporting Information). Masson staining revealed that Ddr1‐WT tumors had abundant aligned collagen fibers at the margins (Figure 2F), while the collagen features (area and orientation) remarkably diminished in Ddr1‐KD group (Figure 2G,H). Simultaneously, tumor‐infiltrating total and activated (CD44^hi^CD62L^lo^) CD8^+^ and CD4^+^ T cells, were more abundant in Ddr1‐KD tumors (Figure 2I), indicating a negative correlation between intratumoral tumor infiltrating lymphocytes (TILs) abundance and DDR1 expression. Immunohistochemical results showed fewer CD4^+^ and CD8^+^ T cells in the core of Ddr1‐WT tumors compared to tumor margins. Conversely, no significant difference was observed in CD4^+^ and CD8^+^ T cell abundance between the core and margin of the tumor in Ddr1‐KD group (Figure 2J). Both stress relaxation and Young's modulus were reduced in Ddr1‐KD group (Figure 2K), indicating decreased tumor stiffness associated with weakened collagen caused by DDR1 knockdown. Taken together, DDR1 promotes collagen barrier strengthen that hinder immune cells infiltration.

### 2.3 ADAM10 Controls Collagen Signaling by Shedding the Ectodomain of DDR1

Tumor cells shed DDR1 ectodomain (DDR1‐ECD) when binding to collagen and thereby promoting collagen alignment.^[^ ^16^ ^]^ However, how DDR1‐dependent signaling is regulated has not been understood. So, we employed additional investigations to elucidate the underlying mechanism regulating the shedding of DDR1‐ECD. First, an in vitro CAFs‐derived 3D ECM model was established to mimic structural and biomolecular features in CRC (Figure S3A,B, Supporting Information).^[^ ^20^ ^]^ Consistent with previous evidence,^[^ ^9^ ^]^ CAFs‐derived ECM displayed a higher degree of collagen alignment (Figure S3C,D, Supporting Information), supporting the involvement of tumor collagen receptors in collagen rearrangement. The drug‐protective assay cultured on different conditions showed that CPT‐11 induced cell growth inhibition was abrogated when MC38 cells on CAFs‐derived 3D ECM compared to where cells were plated on plastic dishes (Figure S3E, Supporting Information).

**Figure 3:**
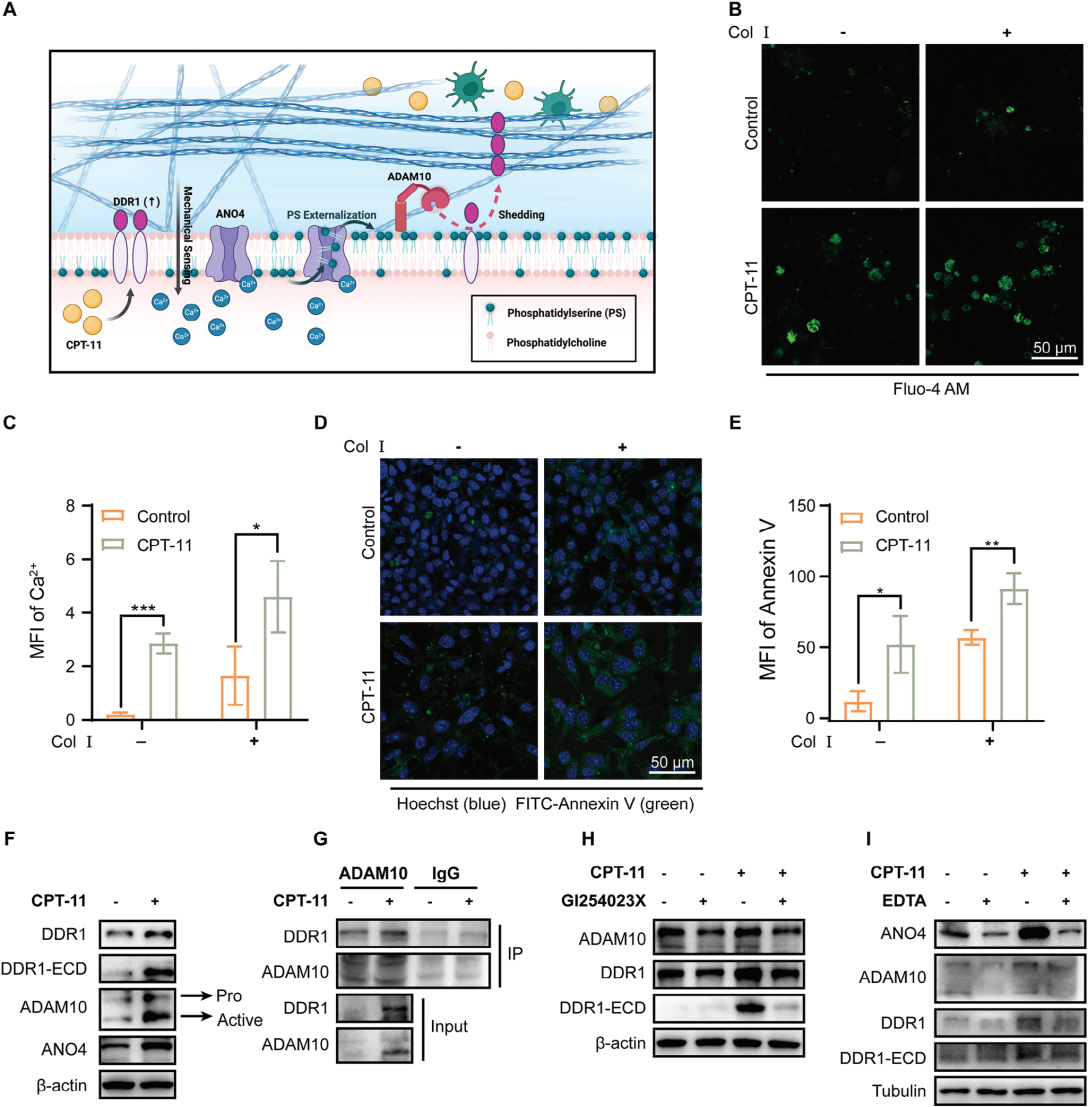
ADAM10 controls collagen signaling by shedding the ectodomain of DDR1. A) Schematic diagram of the mechanism by which DDR1‐ECD is shed. Created with BioRender.com. B–E) Representative images of immunofluorescence for Fluo‐4 AM (B) and Annexin V‐FITC (D) to detect the intracellular calcium ion level and phosphatidylserine (PS) exposure in MC38 cells cultivated on CAFs‐derived ECM, which were pre‐stimulated with or without collagen stimulate for 18 h followed by treatment of CPT‐11 or not for 48 h. Scale bar, 50 µm. The quantitative analyses of mean fluorescence intensity (MFI) were performed by image J, respectively. *n* = 3. Student's t‐test, two tailed. *P < 0.05, **P < 0.01, ***P < 0.001, ****P < 0.0001. F) Immunoblotting analysis of proteins extracted from MC38 cells, which were plated on CAFs‐derived ECM and pre‐stimulated with collagen I (20 µg ml^−1^) for 18 h followed by treatment with CPT‐11 (20 µm) for 48 h. G) Proteins were co‐immunoprecipitated using ADAM10 antibody, the captured ADAM10 and the associated DDR1 proteins were determined using Western blot assays. H,I) Immunoblotting analysis of proteins extracted from MC38 cells, which were plated on CAFs‐derived ECM and pre‐stimulated with collagen I (20 µg ml^−1^) for 18 h followed by treatment with CPT‐11 (20 µm) for 48 h alone, or along with inhibitors, GI254023 (20 µm) for 24 h, EDTA (2 mm) for 3 h, respectively.

Accumulating evidence has shown that plasma membrane dynamics, particularly the surface exposure of phosphatidylserine (PS), are pivotal for exerting sheddase activity.^[^ ^21^ ^]^ Immunofluorescence analysis showed increased intracellular Ca^2+^ and externalization of PS in MC38 cells cultivated on ECM, co‐stimulated by CPT‐11 and collagen (**Figure** **3**B–E). Concurrently, anoctamin 4 (ANO4), a Ca^2+^‐activated phospholipid scramblase,^[^ ^22^ ^]^ was upregulated (Figure 3F). These findings suggest that increased intracellular Ca^2+^ enhanced ANO4 expression, activating its phospholipid scramblase activity, thereby promoting PS externalization to regulate DDR1‐ECD shedding. Subsequently, the focus shifted toward identifying the potential proteinase involved in collagen‐induced DDR1‐ECD shedding.

The impact of ANO family members on A Disintegrin and Metalloproteinases (ADAMs) sheddase activity has been proved.^[^ ^23^ ^]^ ADAM10 and 17 could shed numerous membrane‐bound proteins, releasing their ectodomains.^[^ ^24^ ^]^ Following chemotherapy with CPT‐11, ADAM10 was up‐regulated in CRC cells inoculated on CAFs‐derived ECM, accompanied with an increase in its active form (Figure 3F). Co‐immunoprecipitation of DDR1 with ADAM10 further indicated their presence in a cell surface complex (Figure 3G). Conversely, the appearance of ADAM10 inhibitor GI254023X resulted in a reduction of DDR1 cleavage (Figure 3H). We further reduced intracellular Ca^2+^ levels to explore their impact on ANO4‐mediated ADAM10 sheddase function. The ANO4 elevation induced by chemotherapy was inhibited, leading to decreases in both the truncated subunit of DDR1 and ECD fragments (Figure 3I). These results suggest that elevation of ANO4 enhanced ADAM10 sheddase activity, primarily due to increased PS‐exposure facilitated by Ca^2+^‐activated scramblase.

### 2.4 The Activation of DDR1 Promoted P‐gp Mediated Drug Efflux via the MAPK Pathway in CRC Cells

**Figure 4:**
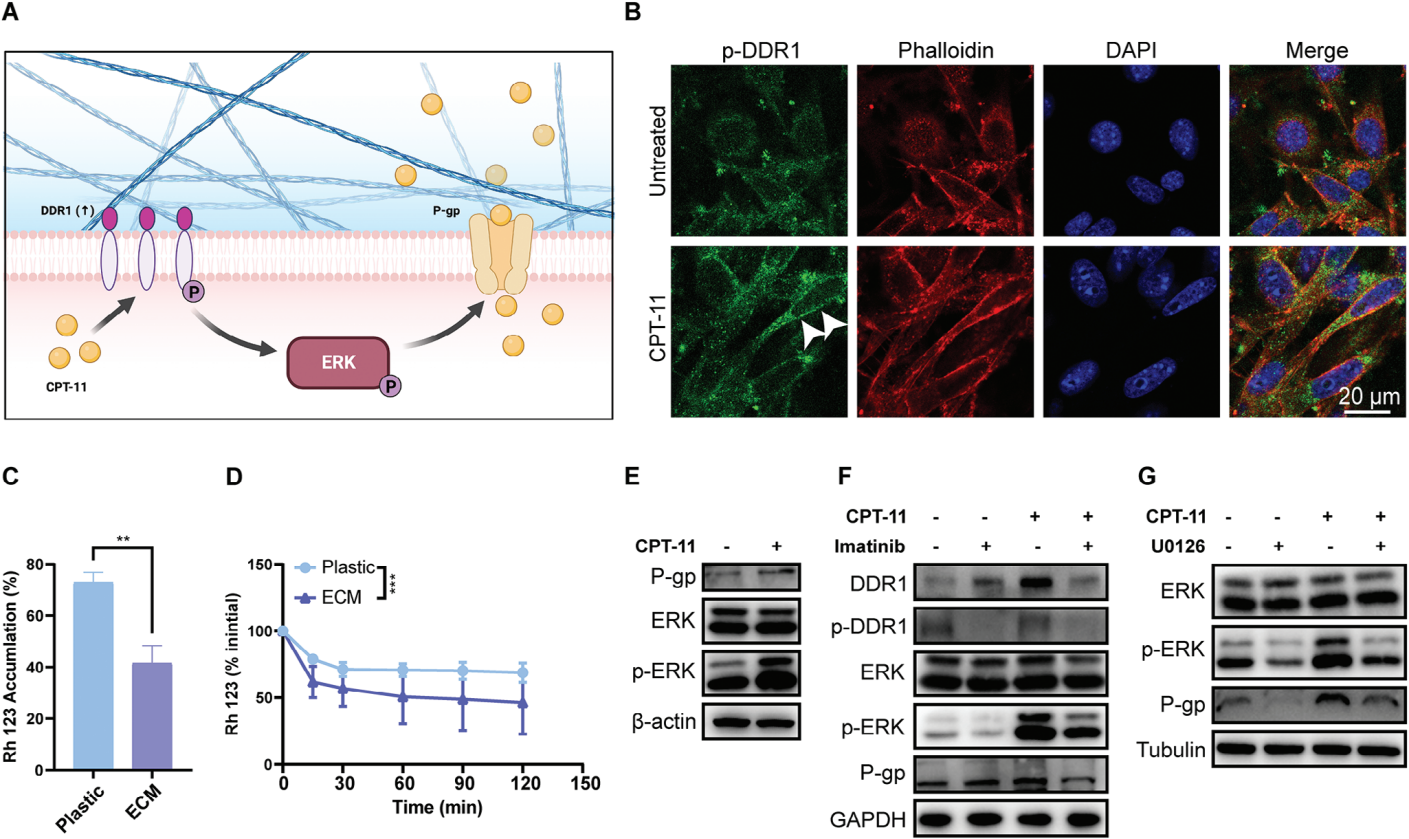
The activation of DDR1 promoted P‐gp mediated drug efflux via the MAPK pathway in CRC cells. A) Schematic diagram of the mechanism by which the intracellular tyrosine kinase activation of DDR1 promotes drug resistance. Created with BioRender.com. B) Representative images of MC38 cells cultivated on CAF‐derived ECMs treated with or without CPT‐11 for 48 h. Immunofluorescence for phospho‐DDR1 (P(Y792) ‐DDR1) (green), F‐actin (red), and nuclei (blue) is shown. White arrows indicate P‐DDR1 cell membrane linear clustering. Scale bar, 20 µm. C,D) The accumulation (C) and efflux (D) curve of fluorescent substrates in MC38 cells cultivated on plastic or CAFs‐derived ECMs. *n* = 3. C). Student's t‐test, two tailed. D). MANOVA Of Repeated Measuring and post‐hoc multiple comparison, two tailed. E–G) Immunoblotting analysis of proteins extracted from MC38 cells, which were plated on CAFs‐derived ECM and pre‐stimulated with collagen I (20 µg mL^−1^) for 18 h followed by treatment with CPT‐11 (20 µm) alone or along with inhibitors, imatinib (20 µm) or U0126 (10 µm), for 48 h.

How tumor cells transmit signals to promote tumor chemotherapy resistance when binding to collagen is still incomplete. On CAFs‐derived ECM, MC38 cells exposed to CPT‐11 showed partial P‐DDR1 distribution into linear clusters, accompanied by F‐actin remodeling (**Figure** **4**B), a phenomenon associated with drug resistance.^[^ ^11^ ^]^ Meanwhile, after DDR1 phosphorylation activation, P‐DDR1 can act as a kinase to catalyze the phosphorylation of downstream target proteins. As CPT‐11 is known substrates for the resistance‐associated ATP binding cassette efflux transporters, to search for the underlying mechanisms of diminished cellular sensitivity, we cultured MC38 cells in different ECM environments using rhodamine 123 replace CPT‐11 as substrates, to assess the intracellular drug levels. The functional transporter assay revealed a reduced intracellular accumulation (Figure 4C) and increased efflux (Figure 4D) of substrates on ECM. P‐glycoprotein (P‐gp) is an important efflux transporter in the ATP binding box transporter family. The overexpression of P‐gp in many tumor cells can pump anti‐tumor drugs out of the cells, thus reducing the concentration of drugs in tumor cells. In addition, P‐gp can also inhibit apoptosis of tumor cells by regulating endogenous and exogenous apoptotic pathways, reducing drug efficacy. We observed that the expression of P‐gp and extracellular regulated protein kinases (ERK) phosphorylation level were significantly increased after collagen stimulated during chemotherapy (Figure 4E). Notably, this upregulation was reversed upon DDR1 tyrosine kinase inhibiting using Imatinib (Figure 4F).^[^ ^25^ ^]^ Additionally, the inhibition of ERK phosphorylation by U0126 effectively counteracted the upregulation of P‐gp (Figure 4G). These suggested that the activation of DDR1 promotes MMDR in CRC through MAPK‐mediated P‐gp overexpression.

### 2.5 Design and Utilization of the Multistage Transportation Nanomedicine

**Figure 5:**
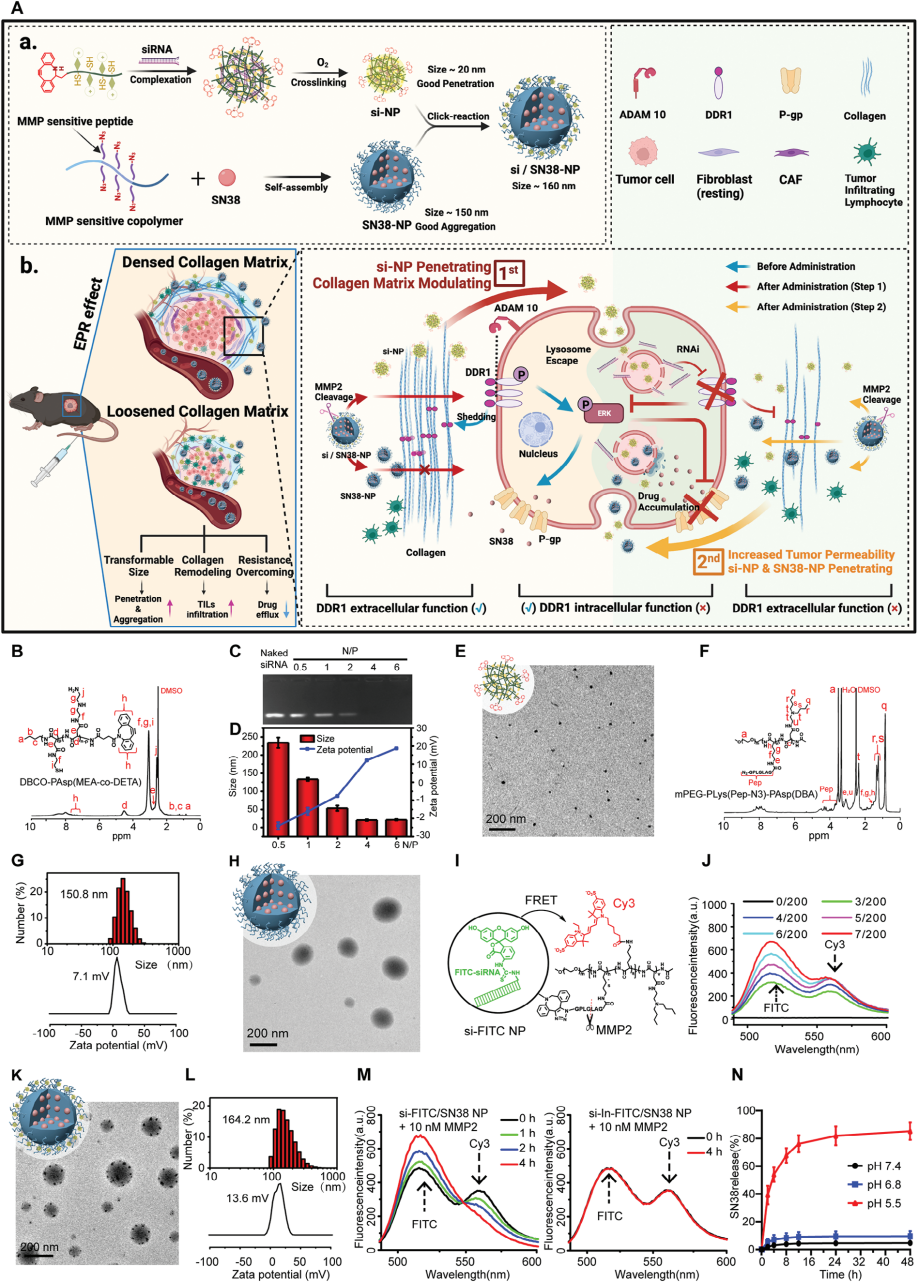
Design and utilization of the multistage transportation nanomedicine. A) Illustration of a multistage nanoparticle delivery of si‐DDR1 and SN38 modulate tumor collagen matrix to overcome matrix‐mediate drug resistance (MMDR). Created with BioRender.com. a) Preparation of the multistage nanoparticle si/SN38‐NP with property of size transformable in response to MMP2. b) Systemically injection of si/SN38‐NP actively targets to tumor ECM modulation, where si/SN38‐NP were activated to liberate si‐NP and SN38‐NP. si‐NP penetrate into tumor cells quickly due to its small size thereby silencing DDR1 and inhibiting collagen alignment (red arrow). Tumor permeability was thus increased to enhance SN38‐NPs penetration and tumoral infiltration of TILs (yellow arrow). At the same time, the knockdown of DDR1 inhibits the expression of P‐gp and enhances the accumulation of drugs in tumor which overcomes MMDR. The role of DDR1 in MMDR before administration is highlighted (blue arrow). B) ^1^H‐NMR spectrum of cationic polymer DBCO‐PAsp (MEA‐*co*‐DETA) in DMSO‐*d*6. C) Electrophoretic mobility of siRNA in agarose gel was evaluated after complexation with DBCO‐PAsp (MEA‐*co*‐DETA) and crosslinking by blowing oxygen at different N/P ratios. D) Particle size and zeta potential of polyplexes si‐NP at different N/P ratios (mean ± SD, *n* = 3). E) TEM image of si‐NP at N/P ratio of 4. Scale bar, 200 µm. F) ^1^H‐NMR spectrum of the MMP2‐sensitive copolymer mPEG‐PLys(Pep‐N3)‐PAsp(DBA) in DMSO‐*d*6. G,H) The size and zeta potential (G) and TEM image (H) of SN38‐NP in PBS (pH 7.4). Scale bar, 200 µm. I) The schematic illustration of FRET effect between the si‐FITC NP loaded with FITC‐siRNA and the SN38‐NP labeled with Cy3 in the interlayer, FITC and Cy3 are used as a FRET pair. J) Fluorescence spectra of the SN38‐NP (200 µg) labeled with Cy3 mixed with the different mass of si‐FITC NP under 490 nm excitation. K,L) TEM image (K), size, and zeta potential (L) of the si/SN38‐NP prepared at the mass ratio of 5/200 (si‐NP / SN38‐NP). Scale bar, 200 µm. M) Fluorescence spectra of MMP2‐sensitive si‐FITC/SN38‐NP and MMP2‐insensitive si‐FITC/In‐SN38‐NP nanoparticles at different time points in solution containing 10 nm MMP2 to mimic the tumor microenvironment. N) SN38 release behaviors from si/SN38‐NP at pH 5.5, 6.8, and 7.4. *n* = 3.

Based on the role of DDR1 in mediating MMDR and immune evasion, the design of nanomedicine targeted to inhibit DDR1 is of great importance for CRC clinical therapy. We propose that by designing a multistage responsive nanomedicine with tunable sizes, carrying DDR1‐siRNA and SN38, enabling tumor ECM active modulation, softening the tumor and blocking cancer cell signaling to overcome MMDR in CRC and achieve a synergistic chemo‐immunotherapy effect (**Figure** **5**A).

First, we synthesized DBCO‐terminated and thiol‐pendant cationic polymer DBCO‐PAsp(MEA‐*co*‐DETA) (Figure S4A, Supporting Information) and employed corresponding characterization (Figure 5B; Figure S4B–D, Supporting Information). The unit numbers of PAsp(DETA) and PAsp(MEA) were determined to be ≈80 and 20, respectively, based on integral calculations of the ^1^H‐NMR peak areas (Figure 5B). Consequently, the molecular weight of the polymer DBCO‐PAsp(MEA‐*co*‐DETA) is calculated to be ≈19.8 kDa (Figure S4E,F, Supporting Information). Small‐sized siRNA nanocomplexes (si‐NP) were prepared using disulfide crosslinking, achieving complete complexation at an N/P ratio of 4. The resulted si‐NP exhibited a uniform spherical morphology with a size of ≈20 nm (Figure 5C–E), suitable for tumor penetration but prone to rapid vivo clearance.^[^ ^26^ ^]^ Moreover, the Raman spectrum of si‐NP confirmed the disulfide‐crosslinked structure (Figure S4G, Supporting Information), enhancing si‐NP stability.^[^ ^27^ ^]^

Subsequently, we synthesized a triblock amphiphilic copolymer mPEG‐PLys(Pep‐N_3_)‐PAsp(DBA) (Figure S5A, Supporting Information). The hydrophilic segment contained a poly(lysinate) block [PLys(Pep‐N_3_)] with MMP2‐sensitive and azide‐terminated pendant peptides, while a pH‐sensitive hydrophobic block poly[N‐(N“,N”‐dibutylethylenediamine)] aspartamide [PAsp(DBA)]. Characterization via ^1^H‐NMR, FTIR, and GPC confirmed successful synthesis (Figure 5F; Figure S5B–G, Supporting Information). The molecular weight of mPEG‐PLys(Pep‐N_3_)‐PAsp(DBA) is ≈27.8 kDa (Figure S5H, Supporting Information), as the degrees of polymerization for PLys(Pep‐N_3_) and PAsp(DBA) were determined to be ≈15 and ≈50, respectively, through integral calculations of the ^1^H‐NMR peak areas and GPC analysis (Figure 5F; Figure S5I, Supporting Information). The copolymer self‐assembled into micelle in PBS (pH 7.4), encapsulating the chemotherapeutic drug SN38 within the core, resulting in pH/MMP2 dual‐responsive SN38‐NP. The particle size was ≈150 nm (Figure 5G,H), which facilitated enhanced tumor accumulation but poor tumor penetration.^[^ ^28^ ^]^

To achieve multistage TME‐responsive size variation for improved tumor penetration while accumulation through ECM, si‐NP was anchored to the interlayer of SN38‐NP to form multistage nanoparticles si/SN38‐NP. The conjugation ratio of si‐NP to SN38‐NP was determined by fluorescence resonance energy transfer (FRET) between Cy3‐labled SN38‐NP and FITC‐labled siRNA within si/SN38 NP (Figure 5I). As the SN38‐NP/siRNA mass ratio increased, the Cy3/FITC fluorescence intensity ratio increased until it exceeded 5/200, signifying maximum conjugation of si/SN38‐NP (Figure 5J; Figure S6A, Supporting Information). The resulting si/SN38‐NP, with a size of 164 nm and a zeta potential of +13 mV, exhibited a spherical morphology with some small‐sized si‐NPs in the interlayer (Figure 5K,L). These results demonstrated the successful interlayer conjugation of si‐NP with SN38‐NP through MMP2‐sensitive peptide.

To assess the multistage delivery capability si/SN38‐NP in response to TME, we compared the drug release behaviors of si/SN38‐NP and its MMP2‐insensitive control group (In‐si/SN38‐NP) in an MMP‐2 environment. Upon MMP‐2 treatment, the FITC representing siRNA gradually increased while the Cy3 representing SN38‐NP decreased in si/SN38‐NP solution. This is attributed to the MMP2‐responsive disassembly of si/SN38‐NP and release of si‐NP, leading to loss of FRET, resulting in enhanced FITC fluorescence. Conversely, In‐si/SN38‐NP, lacked MMP2‐responsive properties, showed no influence on the FITC and Cy3 fluorescence within the nanoparticles due to absence of MMP‐2 responsiveness (Figure 5M; Figure S6B,C, Supporting Information).

Considering that the hydrophobic block PAsp(DBA) of si/SN38‐NP can be protonated and transformed into hydrophilic block under acidic condition,^[^ ^29^ ^]^ resulting in the pH‐sensitive release of SN38. It is evident that SN38 was efficiently released from si/SN38‐NP at pH 5.5, simulating the lysosomal environment (Figure 5N). These results indicated that si/SN38‐NP responds to the MMP2 in TME, allowing small‐sized si‐NP to detach from the nanoparticle surface and penetrate deeply. Additionally, the larger‐sized SN38‐NP was degraded to release the chemotherapeutic drug within the lysosomal environment, achieving multistage drug release and multi‐targeted therapy.

### 2.6 Multistage Delivery si/SN38‐NP Improved Tumor Penetration and Accumulation

**Figure 6:**
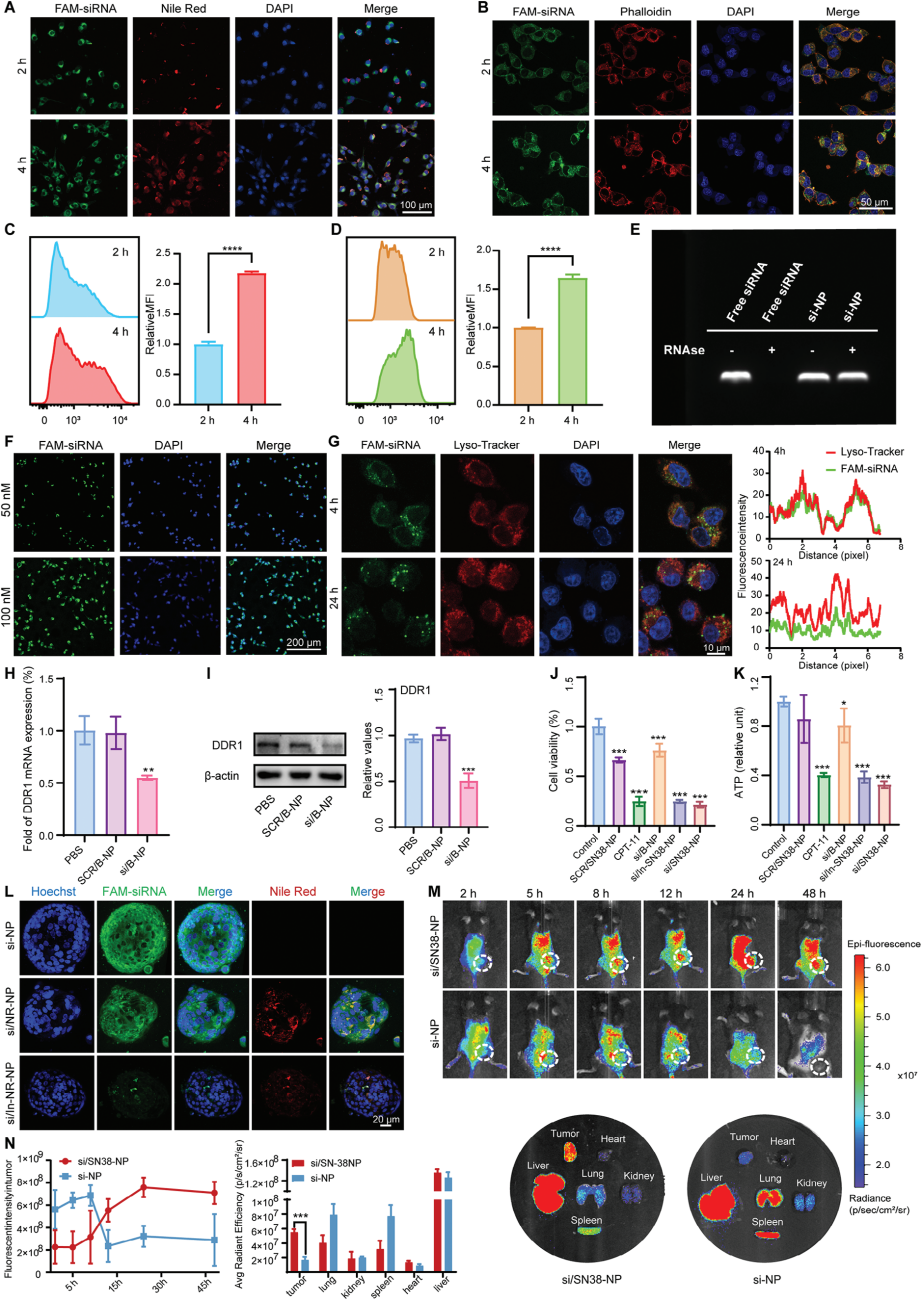
Multistage delivery si/SN38‐NP improved tumor penetration and accumulation. A,B) CLSM images assessed the cellular uptake ability of si/NR‐NP (A) (Scale bar, 100 µm.) and si‐NP (B) (Scale bar, 50 µm.) by MC38 cells. C,D) FCM histograms and MFI values of FAM‐siRNA in MC38 cells after 2 or 4 h incubation with si/NR‐NP (C) and si‐NP (D). Student's t‐test, two tailed. *P < 0.05, **P < 0.01, ***P < 0.001, ****P < 0.0001. E) Protection of siRNA in si‐NP against the digestion by RNAse. F) CLSM images to observe the transfection efficiency of si/B‐NP loaded with different concentrations of FAM‐siRNA after 4 h incubation. Scale bar, 200 µm. G) Subcellular distribution of si/B‐NP (green) in MC38 cells at 4 or 24 h was visualized by CLSM. Nuclei (blue), lysosomes (red). Scale bar, 10 µm. The analysis of the co‐location between FAM‐siRNA and lysosome. H,I) Relative DDR1 mRNA level (G) and DDR1 protein level (H) in MC38 cells after different treatments. The concentration of siRNA used is 100 nm. n = 3. One‐way ANOVA, two tailed. *P < 0.05, **P < 0.01, ***P < 0.001, ****P < 0.0001. J,K) The anti‐tumor effects of si/SN38‐NP on MC38 tumor cells (*n* = 3) J) and on patients derived organoids (PDOs) from chemotherapy resistance CRC patients (*n* = 3 patients) K). One‐way ANOVA, two tailed. *P < 0.05, **P < 0.01, ***P < 0.001, ****P < 0.0001. L) CLSM z‐stack scanning observations of si‐NP and si/NR‐NP and NR‐NP, penetrating into the PDOs after co‐culture for 0.5 h. NR‐NP (Nile Red), FAM‐siRNA (green). Scale bar, 20 µm. M) The fluorescent images of tumor‐bearing mice were collected by IVIS at the indicated time points after receiving intravenous injection of si‐NP and si/SN38‐NP. The ex vivo fluorescent images of tumor tissues and major organs were recorded 48 h after intravenous injection of si‐NP or si/SN38‐NP. N) The quantitative analysis of pixel fluorescence intensity of tumor tissues at the indicated time points and major organs after intravenous injection for 48 h. *n* = 3. Student's t‐test, two tailed, *P < 0.05, **P < 0.01, ***P < 0.001, ****P < 0.0001.

DDR1‐mediated ECM remodeling hinders deep tumor drug delivery. Therefore, the penetrating and DDR1 silencing capabilities of si/SN38‐NP are crucial for the therapeutic efficiency in DDR1‐mediated resistant CRC. We employed Nile Red (NR) as a substitute for SN38 (si/NR‐NP) to simulate nanomedicine delivery. Cellular uptake of nanoscale si/NR‐NP and smaller‐sized si‐NP by MC38 cells was examined using CLSM (**Figure** **6**A,B) and FCM analysis (Figure 6C,D), demonstrating time‐dependent cellular uptake of both nanoparticles. Moreover, siRNA loaded into si‐NP was well protected from degradation by RNAse (Figure 6E). The transfection efficiency of si/B‐NP (the micellar core without SN38 encapsulated) was enhanced with increasing siRNA concentration, reaching nearly 100% at 100 nm (Figure 6F).

Furthermore, low co‐localization ratio between FAM‐siRNA and Lyso‐Tracker indicated effective escape of si/B‐NP from lysosomal entrapment following cellular internalization (Figure 6G). DDR1 silencing efficiency by si/B‐NP was confirmed by qPCR (Figure 6H) and immunoblotting (Figure 6I) analysis in MC38 cells. The cell viability of MC38 cells showed obvious tumor killing effects in the SCR/SN38‐NP (scramble siRNA loaded), si/In‐SN38‐NP and si/SN38‐NP groups (Figure 6J), primarily attributed to the presence of SN38. This aligns with our previous findings that DDR1 did not affect the proliferation of tumor cells in vitro. This conclusion was also evidenced in patients‐derived organoids (PDOs) from chemotherapy‐resistant CRC patients (Figure 6K).

Then the 3D PDOs was utilized to assess penetration. Both si‐NP itself and si‐NP released from si/NR‐NP rapid penetrated into the interior of PDOs within 30 min, benefiting from their size advantage. Meanwhile, the larger‐sized NR‐NP accumulated at the spheroid periphery. In contrast, In‐si/NR‐NP, lacking MMP2 responsiveness, only allowed a minimal amount of drug accumulation inside the spheroids (Figure 6L; Figure S7, Supporting Information), highlighting the role of MMP2‐responsive si‐NP release in deep penetration of si/NR‐NP.

Subsequently, the pharmacokinetics and biodistribution of different nanoparticles were evaluated in CAFs‐mediated resistant mice model post‐intravenous injection. si‐NP rapidly accumulated into the tumor, but was quickly cleared from the body. Conversely, TME‐responsive si/SN38‐NP exhibited prolonged circulation, resulting in higher tumor accumulations and prolonged retention, maintaining a substantial drug concentration at the tumor site even 48 h post‐injection (Figure 6M,N).

### 2.7 In Vivo Antitumor Activity of si/SN38‐NP on CAFs‐Mediated Resistant CRC

**Figure 7:**
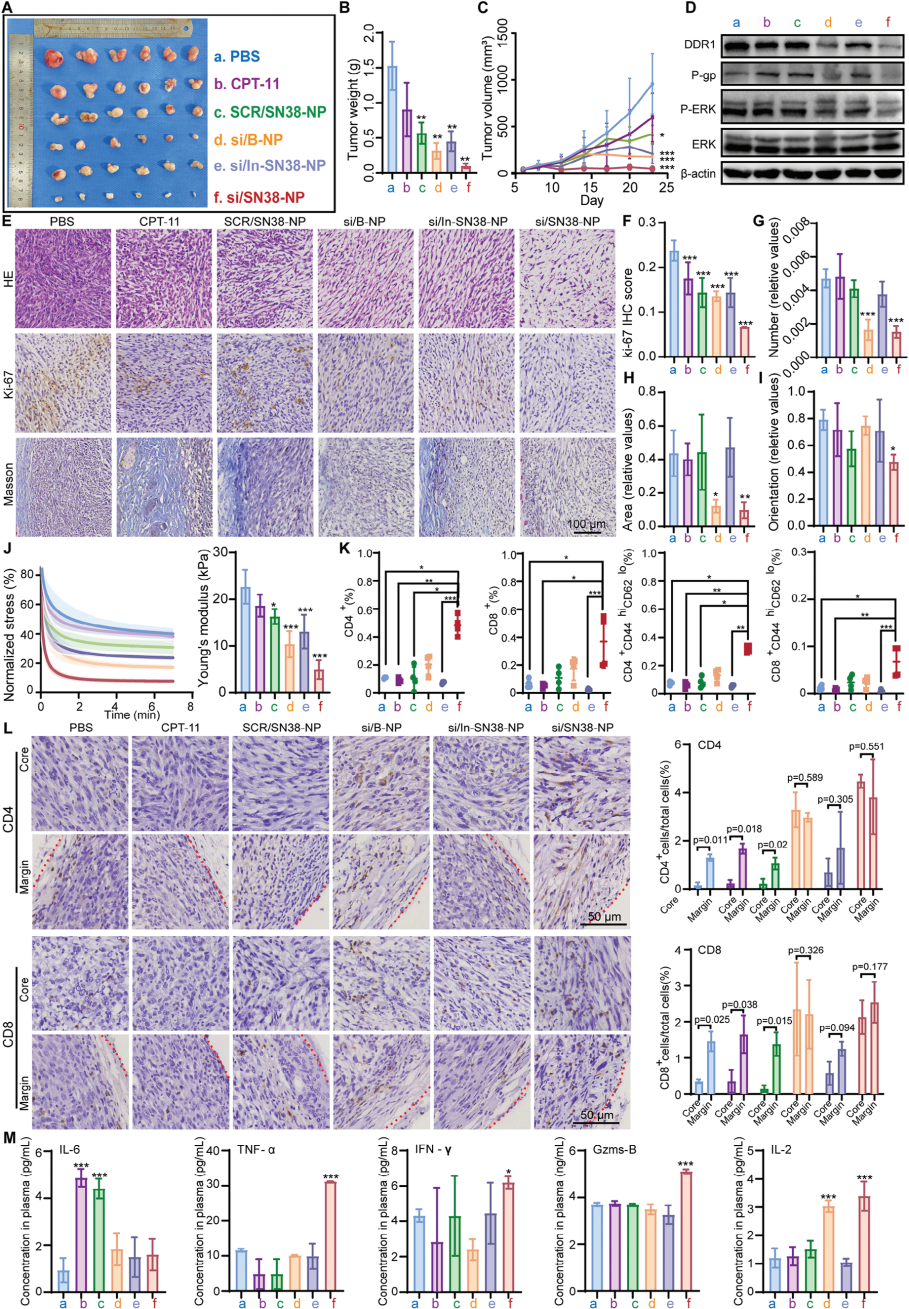
In vivo antitumor activity of si/SN38‐NP on CAFs‐mediated resistant CRC. A) Photographs of the tumors were excised from mice receiving different treatments on day 23. B) Tumor weight of each group at the treatment on day 23. *n* = 6. One‐way ANOVA, two tailed. *P < 0.05, **P < 0.01, ***P < 0.001, ****P < 0.0001. C) Growth curves of the tumors of different treatment groups. *n* = 6. Manva of repeated measuring and post‐hoc multiple comparison, *P < 0.05, **P < 0.01, ***P < 0.001, ****P < 0.0001. D) Immunoblotting of protein extracts from tumor tissues. **E**. The H&E staining, immunohistochemical analysis stained by Ki‐67 protein and Masson staining on the dissected tumor tissue. scale bar, 100 µm. F) Quantitative analysis of Ki‐67 protein expression from IHC image by Image J software. *n* = 3. One‐way ANOVA, two tailed. *P < 0.05, **P < 0.01, ***P < 0.001, ****P < 0.0001. G–I) The collagen features (area, number and orientation) extracted from Masson staining images were quantitatively analyzed by MATLAB software. *n* = 3. One‐way ANOVA, two tailed. *P < 0.05, **P < 0.01, ***P<0.001, ****P<0.0001. J) Stress relaxation of tumor tissues collected from different groups after treatment. Shaded regions represent s.d. of normalized stresses of different samples. Young's modulus of as‐treated tumors was determined. *n* = 3. Student's t‐test, two tailed, *P < 0.05, **P < 0.01, ***P < 0.001, ****P<0.0001. K) Flow cytometry analysis of tumor‐infiltrating total and activated (CD44^hi^CD62L^lo^) CD8^+^ and CD4^+^ T cells in tumor tissues. *n* = 4. One‐way ANOVA, two tailed. *P < 0.05, **P < 0.01, ***P < 0.001, ****P < 0.0001. L) Representative IHC images and corresponding quantification of CD4^+ ^and CD8^+^ T cell infiltrated in core and margin areas of tumor tissues. scale bar, 50 µm. *n* = 3. Student's t‐test, two tailed, *P < 0.05, **P < 0.01, ***P < 0.001, ****P < 0.0001. M) ELISA detection of the levels of IL‐6, TNF‐α, IFN‐γ, Gzms‐B, and IL‐2 in the blood of mice after different treatments. *n* = 3. One‐way ANOVA, two tailed. *P < 0.05, **P < 0.01, ***P < 0.001, ****P < 0.0001.

The antitumor effects of si/SN38‐NP was evaluated in the CAF‐mediated resistant mice model. After different treatments for 18 d, tumors were excised, photographed (**Figure** **7**A), and weighed (Figure 7B). Compared to PBS group, mice treated with chemotherapies (CPT‐11, SCR/SN38‐NP and In‐si/SN38‐NP) exhibited continued tumor growth, while those receiving DDR1‐silencing combined strategy (si/SN38‐NP) achieved nearly complete tumor eradication (Figure 7C). Protein extraction from tumor tissues confirmed DDR1 knockdown by si/SN38‐NP, along with reduced levels of P‐gp and phosphorylated ERK, consistent with cell experiment results (Figure 7D). H&E staining showed significantly lower cell density in si/SN38‐NP group compared to the PBS group (Figure 7E). The most suppressed Ki‐67 also indicated the significant tumor proliferation by si/SN38‐NP (Figure 7F). Collagen number and area as well as the fiber orientation significantly decreased in si/SN38‐NP group (Figure 7G–I), contributing to reduced tumor stiffness and elasticity. We measured tumor stress relaxation and found a significant reduction in elasticity and Young's modulus (Figure 7J) after si/SN38‐NP treatment. These findings demonstrate that si/SN38‐NP effectively reduces tumor stiffness via DDR1‐silencing, weakening ECM‐induced physical barriers.

Given prior speculation that DDR1‐dependent collagen barriers impede immune cell infiltration, we examined the T‐cell proportions in tumor. Tumor‐infiltrating total and activated (CD44^hi^CD62L^lo^) CD8^+^ and CD4^+^ T cells were more abundant in si/SN38‐NP group than others (Figure 7K), and both CD4^+^ and CD8^+^ T cells were evenly distributed in the tumor core and margin. In contrast, PBS, CPT‐11, or SCR/SN38‐NP groups only displayed noticeable enrichment at tumor margins (Figure 7L). Additionally, we detected serum cytokines levels (IL‐6, TNF‐α, IFN‐γ, Gzms‐B, and IL‐2) in all groups (Figure 7M). Increased IL‐6 was found in CPT‐11 and SCR/SN38‐NP groups, associated with chemotherapy resistance and immune suppression.^[^ ^30^ ^]^ In contrast, anti‐tumor immunomodulators (TNF‐α, IFN‐γ, Gzms‐B, and IL‐2) raised in si/SN38‐NP group, signifying immune activation. IL‐2, as reported, can boost CD4^+^ T cells to secrete Gzms‐B to eliminate cancer cells.^[^ ^31^ ^]^

Biological safety of the drug is the primary concern for clinical applications. Thoroughly, the biological safety of si/SN38‐NP was evaluated in vitro and in vivo. First, we tested the cytotoxicity of empty vectors at different concentrations in vitro, and the results showed that there was a difference in cell survival at doses up to 20 µg mL^−1^, which was much higher than the vector dose we used (Figure S8A, Supporting Information). The stability of si/SN38‐NP was studied for 48 h (Figure S8B, Supporting Information), showing si/SN38‐NP was quite stable in serum. To assess the impact of si/SN38‐NP on erythrocyte, hemolysis test was conducted and showed that rarely causes hemolysis (Figure S8C, Supporting Information). Furthermore, the in vivo toxicity of si/SN38‐NP was studied in mice. The body weight of the mice in each groups showed no significant changes during the treatment (Figure S8D,E, Supporting Information). Blood biochemical study showed that key serum biochemical indexes fluctuated slightly in treated mice, but they were all in the normal reference range, indicating that there was no obvious hepatorenal toxicity and heart injury in all treatment groups (Figure S8F, Supporting Information). In addition, after 15 days of various treatments, there was no obvious pathological change in H&E staining of heart, liver, spleen, lung, and kidney tissues in all groups (Figure S8G, Supporting Information). Overall, these results provided compelling evidence for the effectiveness of si/SN38‐NP in CRC treatment, accompanied by a favorable safety profile.

## 3 Discussion and Conclusion

ECM is a highly dynamic structural framework of macromolecules that provides essential biochemical and biomechanical clues for tumor progression.^[^ ^10^ ^]^ Previous studies have revealed the impact of ECM within TME on tumor cell behavior and function, suggesting potential strategies for targeted ECM treatment of cancer.^[^ ^32^ ^]^ Matrix‐mediated drug resistance is prevalent in various tumors, including CRC.^[^ ^33^ ^]^ The cell‐matrix interface promotes chemotherapy resistance by regulating the dormancy of human colon cancer stem cells.^[^ ^34^ ^]^ Modulating ECM stiffness by inhibiting fibroblast contraction and extracellular matrix deposition have been shown to improve drug responses in metastatic CRC.^[^ ^35^ ^]^ However, while most studies have focused on how ECM regulates tumor cell progression and the underlying mechanisms, the reciprocal influence of tumor cells on ECM, which in turn affects TME, has received less attention. Accumulating evidence suggests that collagen, the most abundant component of tumor ECM, plays a crucial role in drug resistance by facilitating ECM remodeling, thereby allowing CRC cells to rapidly adapt to pharmacological treatments.^[^ ^8^ ^]^ Here, we describe a novel mechanism by which CRC cells develop tolerance to chemotherapeutic drug CPT‐11 through dynamic interactions with collagen, and demonstrate that targeting cell surface receptor DDR1 in tumors to reshape the collagen barrier is a viable way to improve drug penetration to tumors.

Collagen architecture at tumor‐stroma boundary directs tumor cell invasion.^[^ ^36^ ^]^ Therefore, quantitative analysis of collagen signatures at IM and CT of tumors helps to fully understand the spatial heterogeneity of TME, which can not only reveal how tumors develop, but also accelerate the development of novel cancer therapies. In our study, we provide evidence for the role of collagen‐rich matrices in the adaptive phase of tolerance to chemotherapies. Based on Masson staining images, the detailed features of collagen were obtained. Then we constructed an innovative interregional variation CS_IM/CT_ for quantitative analysis of collagen barrier and further analyzed its correlation with DDR1 expression level, confirming that DDR1 regulates MMDR of CRC through collagen remodeling. To our knowledge, this is the first study linking quantitative alterations in collagen features with DDR1.

Existing models suggest that the kinase activity of DDR1 plays a major role in tumorigenesis.^[^ ^37^ ^]^ However, other studies have shown that its extracellular domain (DDR1‐ECD) promoted cancer cells migration or immune excluded independent with its kinase activity.^[^ ^16^, ^38^ ^]^ Whether DDR1 mainly depends on intracellular tyrosine kinase domain or ECD plays a role in colorectal cancer drug resistance is still controversial. How the extracellular domain is shed after binding with collagen remains unclear. We found that after the extracellular domain of DDR1 binds to collagen, ECM stiffness related mechanical sensing can increase the intracellular calcium ion level and up‐regulate the expression of Ca^2+^ activated chloride ion channel ANO4. Meanwhile, ANO4, as a Ca^2+^ activated phospholipid recombinase, can promote the externalization of PS and enhance the cleavage of ADAM10 on the extracellular domain of DDR1. The shed ECD can enhance the binding of collagen in ECM and make collagen fibers compact and form a collagen barrier, which makes it difficult for chemotherapy drugs to penetrate and prevents the infiltration of immune cells. The binding of collagen on the other side will activate tyrosine kinase activity in DDR1 cells, promote the phosphorylation of ERK, and lead to increased expression of P‐gp, the drug transporter, resulting in drug resistance. Our results fill in the gaps in the detailed mechanisms by which DDR1 promotes colorectal cancer MMDR.

DDR1 is becoming an attractive target for anti‐cancer therapies as its role in regulating MMDR.^[^ ^39^ ^]^ The existing matrix modulation methods have not yet achieved satisfactory results. On the one hand, the treatment of regulating ECM through matrix degradation pathways may lead to the release of cytokines and inflammatory factors, promoting tumor progression. On the other hand, the degradation of ECM may lead to tumor invasion and metastasis.^[^ ^8^ ^]^ In addition, the reason why some treatments targeting integrin have not made progress may be due to its expression on immune cells. Inhibition of integrin may affect immune surveillance.^[^ ^40^ ^]^ Addressing the shortcomings of the prior approaches of ECM modulation, DDR1 have some advantages. First, DDR1 mainly leads to MMDR by regulating the arrangement of collagen fibers and the expression of P‐gp efflux proteins, which is different from the principle of mere degradation of matrix. Furthermore, inhibiting DDR1 could boost tumor immunity. As well as DDR1 is relatively less expressed on immune cells, making it a more suitable specific target. Inhibition of DDR1 by nilotinib, dasatinib, or other unapproved inhibitors has been shown to reduce tumorigenicity, aggressiveness, or metastasis of several cancers.^[^ ^41^ ^]^

The development of inhibitors and antibodies against DDR1 is accelerating, but still have limitations. The macromolecular antibody drugs are difficult to develop and expensive. Current small‐molecule DDR1 inhibitors target their kinase domains non‐specifically and have no effect on kinase‐independent ECD activity. RNAi therapy has the characteristics of wide range of potential drug targets, no genotoxicity, strong persistence, faster and higher development success rate and relatively low manufacturing cost, enabling precise and personalized therapies. As RNAi drugs gain more attention in the market, the main challenges will be targets selection and delivery technology.^[^ ^42^ ^]^

siRNA drugs need to overcome the tumor matrix barrier, achieve cell endocytosis and lysosome escape, and avoid degradation under nuclease action. The precise design of size, structure and surface modification of nanocarriers is the important factors for the successful delivery of siRNA drugs.^[^ ^43^ ^]^ As the particle size increases, the blood circulation time and tumor aggregation are enhanced, and the optimal particle size is around 100–160 nm. However, large particle did not significantly improve efficacy because its poorer tumor penetration than small particle.^[^ ^28^ ^]^ In order to target the dense collagen, optimizing the size of nanomaterials to balance the penetration and accumulation ability of nanomaterials is the key to improve therapeutic effectiveness. Therefore, we used a multistage system si/SN38‐NP with transformable size triggered by high expression of MMP2 in the TME, which were activated to liberate si NP (≈20 nm) and SN38‐NP (≈150 nm) from ≈160 nm nanoparticles. si‐NP are more likely to penatrate into the tumor stroma, thereby silencing DDR1 and modulating collagen barrier. Tumor pemeability was thus increased to enhance the difussion of SN38 NPs and tumoral infiltration of TILs. Meanwhile, the knockdown of DDR1 inhibits the expression of P‐gp and reduces drug efflux. Animal experiments have confirmed that this nano delivery system can efficiently knock down DDR1, remodel ECM and significantly improve the anti‐tumor effect.

In summary, we described a novel mechanism of chemotherapy resistance resulting from dynamic interactions between tumor cells and collagen. For the first time, we validated in CRC that DDR1 drives tumor progression via both intracellular kinase activity and ECD routes. Upon this, we designed a TME‐responsive multistage nanomedicine (si/SN38‐NP) to reverse MMDR by suppressing DDR1. The simultaneous good penetration and accumulation ability of this nanomedicine is achieved by its transformable size. The nano‐platform proposed in this study may be applied in the future design of other therapeutic drugs to disrupt the collagen barrier for high‐efficient drug delivery.

## 4 Experimental Section

### Materials

Dulbecco's Modified Eagle Medium (DMEM) (Cat#C11995500BT) and Opti‐MEM (Cat# 31 985 070) were purchased from Gibco, Thermo Fisher Scientific Co., Ltd (Massachusetts, USA). Fetal bovine serum (FBS) was purchased from Procell Life Science & Technology Co., Ltd (Wuhan, China). GI254023X (Cat#HY‐19956), Imatinib Mesylate (HY‐50946), U0126‐EtOH (HY‐12031) were purchased from MedChemExpress Co., Ltd (New Jersey, USA). EDTA (Cat#E1170), L‐Ascorbic Acid Sodium Salt (Cat#134‐03‐2), Rhodamine 123 (Cat#62669‐70‐9), Hoechst 33 342 Stain solution (ready‐to‐use) (Cat#C0030), DAPI solution (ready‐to‐use) (Cat#C0065), Mounting Medium, antifading (Cat#S2100), Collagenase I (Cat#C8140) were procured from Solarbio Sciences & Technology Co., Ltd (Beijing, China). Triton‐NH_4_OH extraction buffer (Cat#PS0005), Masson Trichrome Kit (Cat#DC0033) and Harris hematoxylin (H&E) solution (Cat#DH0006) were purchased from Leagene biotechnology Co., Ltd (Beijing, China). Alexa Fluor 594 Phalloidin (Cat# A12381) was purchased from Sigma‐Aldrich Co., Inc. (St. Louis, USA). Universal Kit (Mouse/Rabbit polymer test system) (Cat# PV‐6000), DAB Kit (Cat#ZLI‐9017) were purchased from OriGene (Beijing, China). Collagen Type I Rat Tail (Cat#354 236) and Matrigel (Cat#345 230) were purchased from Corning Co., Ltd (New York, USA). Fluo‐4 AM (Cat# MA0196) was purchased from Meilunbio Co., Ltd (Dalian, China). BBproExtra RIPA lysis buffer (Cat#BB‐3201) was purchased from BestBio Co., Ltd (Shanghai, China). Immunoprecipitation Kit (HRP Labeled Protein A) (Cat#PK10007) was purchased from Proteintech (Wuhan, China). Lyso‐tracker Red (Cat#C1046) was purchased from Beyotime Biotechnology Co., Ltd (Shanghai, China). FastPure Cell/Tissue Total RNA Isolation Kit V2 (Cat#RC112‐01), HiScript III All‐in‐one RT SuperMix perfect for Qpcr (Cat#R333) and Taq Pro Universal SYBR qPCR Master Mix (Cat#Q712) were purchased from Vazyme Co., Ltd (Nanjing, China). Tumor Tissue Digestion Solution (Cat# K601003), Colorectal Cancer Organoid Complete Medium (Cat#K2103‐CR) and Organoid Viability ATP Assay Kit (Cat#E238003) were purchased from BioGenous Co., Ltd (Shangdong, China). Mouse IFN‐gamma ELISA Kit (Cat#GEM0006), Mouse IL‐6 ELISA Kit (Cat#GEM0001) and Mouse TNF‐alpha ELISA Kit (Cat#GEM0004) were purchased from Servicebio Co., Ltd (Wuhan, China). Mouse IL‐2 ELISA Kit (Cat#EK202/2) was purchased from Multi Science Co., Ltd (Hangzhou, China). Mouse Gzms‐B ELISA Kit (Cat#F2339‐B) was purchased from FANKEW Co., Ltd (Shanghai, China). Cell Counting Kit‐8 (Cat#C6005) were purchased from New Cell & Molecular Biotech Co., Ltd (Suzhou, China). mPEG‐NH2 (Mn = 2 kDa), n‐butylamine, 2‐mercaptoethylamine (MEA), diethylenetriamine (DETA), acetyl chloride, N,N‐dibutylethylenediamine (DBA), 1‐(3‐dimethylaminopropyl)−3‐ethylcarbodiimide hydrochloride (EDC), N‐hydroxy succinimide (NHS), and HBr (33% in acetic acid) were purchased from Aladdin Ltd (Shanghai, China). Irinotecan hydrochloride trihydrate (Cat#BD2019), Ltd (Shanghai, China).β‐benzyl‐L‐aspartate‐N‐carboxyanhydride (BLA‐NCA), Nɛ‐carbobenzoxy‐L‐lysine‐N‐carboxyanhydride [Lys(Cbz)‐NCA], dibenzocyclooctyne‐N‐hydroxysuccinimidyl ester (DBCO‐NHS), and 7‐ethyl‐10‐hydroxycamptothecin (SN38) were procured from Bide Pharmatech company (Shanghai, China). The MMP2‐sensitive peptide (N3‐GPLGLAG) and the MMP2‐insensitive peptide (N3‐GLALGPG) were obtained from Haode Peptide Company (Wuhan, China). Dimethyl ether, methanol, DCM, DMSO, DMF, and other general chemical reagents were purchased from Guangzhou Chemical Reagent Company (Guangzhou, China).

### Cell Lines

MC38 (Mouse Colon Cancer Cells) and NIH‐3T3 (Mouse Embryonic Fibroblast Cells) were purchased from the American Type Culture Collection (ATCC) and was maintained in DMEM containing 10% FBS and 1% penicillin/streptomycin solution in a humidified atmosphere containing 5% CO_2_ at 37 °C.

### Clinical Sample

This study was approved by the Medical Ethics committee of Nanfang Hospital of Southern Medical University (Approval NFEC‐2023‐247), with all participants providing informed consent. The clinical data of 94 patients who received curative resections between November 2009 and April 2011 at Nanfang Hospital, Southern Medical University were retrospectively analyzed. The inclusion criteria were as follows: 1) age between 18 and 80 years; 2) histologically confirmed colorectal cancer;3) American Joint Committee on Cancer (AJCC) stageII‐III. The exclusion criteria were as follows: 1) pregnancy; 2) concurrent presence of other tumors. Patients were divided into two groups based on whether DFS exceeded 5 years. There were 48 patients with DFS ≥ 5 years and 46 patients with DFS < 5 year. The baseline information included patient demographics (age, sex), AJCC stage, clinical T stage (cT), clinical N stage (cN), histology type and differentiation. Follow‐up duration was measured from the time of surgery to the last follow‐up date, and information regarding the survival status at the last follow‐up was collected.

### Immunohistochemistry and H&E Staining

Five micrometers sections were deparaffinized and rehydrated in PBS, and subjected to heat‐induced antigen retrieval in 10 mm citrate buffer (pH 6.0). Endogenous peroxidases were inhibited by incubation with 3%H_2_O_2_. Subsequently, the sections were blocked for 1 h at RT with goat serum. The primary antibody was incubated overnight at 4 °C. Tissues were washed before incubating the secondary antibody for 30 min at RT. DAB was prepared and used to stain the sample, which was then examine under a microscope. After staining, tissues were washed in distilled water and stained with Hematoxylin dye. After dehydration, coverslips were mounted on the slides. Samples were imaged by Slide scanner (Shengqiang Technology, China).

The immunohistochemical staining score and percentages of positive cells were quantified by Image J software. The cutoff for determining low or high expression was determined by the optimal cutoff value obtained from ROC curve.

For H&E staining, a H&E solution Kit was used following the manufacturer's instructions.

### Fibrillar Collagen Imaging and Collagen Feature Extraction

Masson trichrome staining was conducted to stain collagen content in tumors. A Masson Trichrome Kit was used following the supplier's recommended protocol. A total of 142 collagen features were automatically extracted from the Masson staining images by MATLAB 2018b (Mathworks, Natick, MA, USA). Subsequently, LASSO regression was used to select the potential predictive features from the 142 collagen features, and then the integrated collagen score of the IM divided by the CT (CS_IM‐CT_) was established.

### Activating Fibroblasts into CAFs

MC38 cells were cultured on 100 mm dishes. When the density reached 70%, the medium was changed to serum‐free medium and starved for 18 h. Then the cell medium was collected, centrifuged for 4000 g × 10 min, and the supernatant was taken and filtered by a 0.45 cell filter, which was used as the tumor conditional medium (TCM).

NIH‐3T3 were seeded in wells of 6‐well plates at a density of 5 × 10^4^ cells per well and incubated overnight, and then treated with a mixture of complete medium and tumor conditioned medium (TCM) from MC38 cells (1:1) for 24 or 48 h. Then the cells were collected to validate the activation of CAFs.

### Immunofluorescence

Cells were fixed with 4% paraformaldehyde for 20 min, permeabilized with 0.1% Triton X‐100 for 10 min, blocked for 1 h at room temperature (RT) with 5%BSA, and incubated overnight with primary antibodies at 4 °C. The antibodies and their working dilutions are listed in Table S1 (Supporting Information). Cells were washed and incubated with secondary antibodies in 1%BSA for 30 min at 37 °C. Phalloidin (1:100) was used to stain F‐Actin (optional), DAPI or Hoechst33342 were used to stain the nucleus (optional). Then the coverslips were mounted on slides. Images were photographed by CSLM (Zeiss LS980).

### Immunoblot and Immunoprecipitation

Whole‐cell lysates were prepared using BBproExtra RIPA lysis buffer. Proteins were separated by SDS‐PAGE and transferred onto PVDF membranes for immunoblot analysis. The membranes were incubated with the primary antibody overnight at 4 °C, washed, and then incubated with the peroxidase‐conjugated secondary antibody. Blots were developed with a chemiluminescence system (Tanon, China).

For immunoprecipitation (IP) assays, an Immunoprecipitation Kit (HRP Labeled Protein A) was used following the manufacturer recommendations. Cells were lysed and then incubated overnight at 4 °C with continuous rocking in lysis buffer containing Protein G Sepharose beads and anti‐ADAM10 antibody. The beads were then washed 3 times with washing buffer supplemented with protease and phosphatase inhibitors. Afterward, the precipitated complex was eluted with Elution buffer. The IP products were separated using SDS–PAGE and subjected to immunoblot analysis.

### siRNA and Lentiviral Genes Transduction

siRNA was purchased from Youming Biological Technology Co., LTD (Guangzhou, China) and the siRNA sequences are listed in Table S2 (Supporting Information). Transfection was performed by Hieff Trans in vitro siRNA/miRNA Transfection Reagent.

Lentiviral siRNAs targeting DDR1 were constructed by Genechem Medical Technology Co., LTD (Shanghai, China). MC38 Cell lines stably expressing Ddr1‐KD were generated through lentiviral LV‐Ddr1‐RNAi infection (48 h) followed by a two‐week puromycin selection (final concentration puromycin: 3 µg ml^−1^).

### Mouse Experiments

C57BL/6 mice (male, 4–6 weeks old) and BALB/c Nude mice (male, 4–6 weeks old) were purchased from the Experimental Animal Center of Southern Medical University. All animal experiments were performed according to the protocols approved by the Institutional Laboratory Animal Ethics Committee of Nanfang Hospital, Southern Medical University (Approval IACUC‐LAC‐20221116‐001).

For co‐injection experiments in C57BL/6 mice, mouse models were established by subcutaneously injecting MC38 cells (1 × 10^6^) alone or in combination of TCM‐induced CAFs (2 × 10^6^) into the right flank of the mice. Tumor size was calculated using the formula: volume = width^2^ ×length/2. Tumor sizes at the endpoint were compared between groups. Mice were sacrificed at the endpoint or when the longest tumor diameter of tumors reached 1.5 cm.

In order to transplant tumors from immunodeficient hosts to immunocompetent hosts, tumor cells were initially inoculated into BALB/c nude mice. When the tumor volume reaches exceeded 1000 mm^3^ (≈20 days after the initial inoculation), ≈60 mg tumor blocks were transplanted into wild‐type C57BL/6 mice.

### Mechanical Analysis of Tumor

Mechanical analysis of tumor samples was conducted through a series of tests, including uniaxial compression and stress relaxation, using a dynamic mechanical analyzer (TA‐DMA850, TA Instruments). To facilitate these tests, tumor samples were carefully positioned between parallel stainless‐steel plates with a diameter of 1.5 cm. An excess of buffer solution was employed to ensure that the samples remained adequately hydrated throughout the duration of the experiments. All mechanical tests were conducted at a controlled temperature of 30 °C. For the uniaxial compression tests, aimed at quantifying the Young's moduli of the tumors, the tumor samples were pre‐loaded with a compressive force of 0.05 N. Subsequently, they were subjected to a controlled compressive strain at a constant rate of 5% per minute. The average Young's moduli for both control and treated tumors were determined by analyzing the stress‐strain curves within the linear viscoelastic regions, which were observed at compression levels below 10%.

In the case of stress relaxation tests, designed to assess the viscoelastic properties of the tumors, the tumor samples were also pre‐loaded with a compressive force of 0.05 N. These samples were then subjected to a uniaxial compressive step strain of 3%. The generated compressive stress was continuously monitored for a duration of 420 s.

### Flow Cytometry

Mouse tumor sample were harvested, cut into pieces with a diameter of nearly 2 mm, diameter pieces and digested to obtain a single‐cell suspension. The suspended cells were incubated with fluorochrome‐conjugated antibodies for 30 min. The samples were then analyzed using the FACS Celesta flow cytometer (BD). Data were processed using FlowJo (Treestar).

### CAFs‐Derived 3D ECM

3D decellularized ECMs were generated as previously described.^[^ ^32^ ^]^ NIH‐3T3 cells were inoculated in a gelatin coated dish and cultured in a mixture of complete medium and tumor conditioned medium (TCM) from MC38 cells (1:1) for 5 days, treated with 50 mg mL^−1^ L‐Ascorbic Acid Sodium Salt every 48 h. Then the cells were decellularized using preheated Triton‐NH_4_OH extraction buffers.

### Matrix‐Mediated Drug Resistance Assay

MC38 cells were seeded on plastic or CAFs‐derived 3D ECM, cultured at 37 °C in 5% CO_2_ for 24 h, and then cultured in a complete medium with CPT‐11 (20 µm) for further 24, 48, or 72 h. To test the cell proliferation, the total number of cells was counted with an automatic counter (Thermo Fisher Scientific).

### ABC Transporter Function Assay

**1) Drug accumulation assay**: Fluorescent substrate Rhodamine 123 (Rho123) was the substrate of ABCB1 and ABCG2. According to the fluorescence characteristics of the drug, Rhodamine 123 was used to replace CPT‐1 for intracellular drug accumulation experiment. MC38 cells per well (2 × 10^5^) were cultured on Plastic and CAFs‐derived 3D ECM six‐well plates for 24 h, and Rho123 (5 µm) was added into the cell medium and cultured for 30 min. The cells were digested and washed 3 times with cold PBS. Finally, the cells were suspended with ice PBS and placed on ice for immediate flow analysis.
**2) Drug efflux assay**: MC38 cells per well (2 × 10^5^) were cultured on Plastic and CAFs‐derived 3D ECM six‐well plates for 24 h, and Rho123 (5 µm) was added to the cell medium and cultured for 30 min. Washed with PBS for 3 times to fully clean Rho123, and then replaced with medium for further culture. Cells were digested and collected at incubation times of 0, 15, 30, 60, 90, and 120 min, respectively. The cells were suspended with cold PBS and placed on ice for immediate flow analysis.

### Synthesis of PBLA

PBLA was synthesized through a ring‐opening polymerization of BLA‐NCA, initiated by *n*‐butylamine. In brief, 3 g of BLA‐NCA (12 mmol) and 12 µL of *n*‐butylamine (0.12 mmol) were dissolved in a mixture of DMF (3 mL) and anhydrous dichloromethane (20 mL) under an argon atmosphere. The solution was stirred for 2 days at 35 °C, concentrated using rotary evaporation, precipitated in cold diethyl ether, filtered, and then vacuum‐dried, resulting in white powder of PBLA. The successful synthesis of the polymer PBLA was confirmed by analyzing the ^1^H‐NMR spectrum.

### Synthesis of DBCO‐PBLA

To obtain DBCO‐PBLA, 1 g of PBLA (0.048 mmol) and 0.1 g of DBCO‐NHS (0.25 mmol) were dissolved in 5 mL of anhydrous CHCl_3_. The solution was stirred for 48 h, followed by dialysis (MWCO: 1 kDa) against methanol for 48 h. After dialysis, the solution was evaporated and then vacuum‐dried, resulting in the synthesis of DBCO‐PBLA. The successful synthesis of the polymer DBCO‐PBLA was confirmed by analyzing the ^1^H‐NMR spectrum.

### Synthesis of DBCO‐PAsp (MEA‐co‐DETA)

DBCO‐PBLA (0.024 mmol) (0.5 g) and 37 mg of MEA (0.48 mmol) were dissolved in 2 mL of anhydrous DMSO. The solution was stirred for 24 h at room temperature. Afterward, 2 g of DETA (19.4 mmol) were added to the above solution. The mixed solution was stirred for an additional 24 h at 35 °C, followed by dialysis (MWCO: 1 kDa) against methanol for 48 h. The purified solution was then subjected to rotary evaporation to remove the solvent and subsequently vacuum‐dried, resulting in the polymer DBCO‐PAsp(MEA‐co‐DETA). The successful synthesis of the polymer DBCO‐PAsp(MEA‐co‐DETA) was confirmed by analyzing the ^1^H‐NMR spectrum.

### Synthesis of mPEG‐PLys(Cbz)‐PBLA

In order to synthesize the triblock copolymer mPEG‐PLys(Cbz)‐PBLA, a ring‐opening polymerization was conducted by reacting Lys(Cbz)‐NCA and BLA‐NCA with APEG‐NH_2_ as a macroinitiator. Initially, 1.0 g of mPEG‐NH_2_ (0.5 mmol) was dried under vacuum at 75 °C for 1.5 h in a 100 mL Schlenk flask. Once the temperature reached 35 °C, a reaction system was prepared under an argon atmosphere by adding 30 mL of anhydrous dichloromethane and 2.3 g of Lys(Cbz)‐NCA (7.5 mmol) dissolved in 2 mL of anhydrous DMF. The polymerization process was allowed to proceed for 2 days. Following that, 6.23 g of BLA‐NCA (25 mmol) dissolved in 6 mL of anhydrous DMF was added to the reaction system under an argon atmosphere. The polymerization was continued for an additional 2 days. The resulting reaction solution was then precipitated in diethyl ether to obtain the polymerization product. Finally, the polymer was dissolved in 10 mL of anhydrous dichloromethane. To block the terminal amino group of the polymer, 107 µL of acetyl chloride (1.5 mmol) was added. After 4 h of reaction, the solution was precipitated in diethyl ether, filtered, washed thrice with diethyl ether, and dried under vacuum. The successful synthesis of the polymer mPEG‐PLys(Cbz)‐PBLA was confirmed by the ^1^H‐NMR result.

### Synthesis of mPEG‐PLys(Cbz)‐PAsp(DBA)

The copolymer mPEG‐PLys(Cbz)‐PAsp(DBA) was synthesized through the aminolysis reaction of mPEG‐PLys(Cbz)‐PBLA with DBA. Briefly, 2.0 g of mPEG‐PLys(Cbz)‐PBLA (0.12 mmol) was dissolved in 30 mL of anhydrous DMF under an argon atmosphere. Subsequently, 5.2 g of DBA (30 mmol) were added to the Schlenk flasks. The aminolysis reaction was carried out at 35 °C for 12 h. After the reaction, the mixture was dialyzed (MWCO: 1 kDa) against methanol for 48 h remove excess DBA. It was then evaporated, precipitated in diethyl ether, and dried under vacuum. The successful synthesis of the polymer mPEG‐PLys(Cbz)‐PAsp(DBA) was confirmed by the ^1^H‐NMR result.

### Synthesis of mPEG‐PLys‐PAsp(DBA)

mPEG‐PLys(Cbz)‐PAsp(DBA) (1 g) was dissolved in 10 mL of trifluoroacetic acid (TFA). Subsequently, 4 mL of 33% hydrobromic acid in acetic acid was added, and the reaction mixture was allowed to react for 4 h at room temperature. The resulting solution was then subjected to dialysis (MWCO: 1 kDa) against methanol for 48 h. Afterward, the solution was evaporated and dried under vacuum to obtain the product mPEG‐PLys‐PAsp(DBA). The successful synthesis of the polymer mPEG‐PLys‐PAsp(DBA) was confirmed by analyzing the ^1^H‐NMR spectrum.

### Synthesis of mPEG‐Plys (Pep‐N_3_)‐PAsp(DBA)

The MMP2‐sensitive copolymer mPEG‐PLys(Pep‐N_3_)‐PAsp(DBA) was synthesized through an amidation reaction between mPEG‐PLys‐PAsp(DBA) and the peptide (N_3_‐GPLGLAG), utilizing EDC and NHS as coupling reagents. Briefly, 0.2 g of the peptide (0.29 mmol), 0.17 g of EDC (0.87 mmol), and 0.1 g of NHS (0.87 mmol) were dissolved in 2 mL of DMSO. After reacting for 2 h, 0.42 g of mPEG‐PLys‐PAsp (DBA) (0.024 mmol) dissolved in 2 mL of DMSO was added to the mixture. The solution was stirred for 48 h at 35 °C, followed by dialysis (MWCO: 3.5 kDa) against methanol for 48 h. After dialysis, the solution was evaporated and the resulting product was dried under vacuum. The successful synthesis of the polymer mPEG‐Plys (Pep‐N_3_)‐PAsp(DBA) was confirmed by analyzing the ^1^H‐NMR spectrum. The MMP2‐insensitive copolymer was synthesized following the same steps as described above with the exception of substituting the MMP2‐sensitive peptide (N_3_‐GPLGLAG) with the MMP2‐insensitive peptide (N_3_‐GLALGPG).

### Preparation of si‐NP

The cationic polymer DBCO‐PAsp(MEA‐co‐DETA) was dissolved in PBS (pH 7.4) at a concentration of 1 mg mL^−1^. Subsequently, the polymer solution was mixed with an siRNA solution (1 mg mL^−1^ in DEPC water) at various N/P ratios. After allowing the mixture to stand undisturbed for 15 min, it was further subjected to oxygen bubbling for 30 min, resulting in the formation of disulfide‐cross‐linked polyplex si‐NP. The optimal N/P ratio of the polyplex was determined through measurements including agarose gel retardation assay, as well as size and zeta potential analysis.

### Agarose Gel Retardation Assay

The si‐NP solutions with various N/P ratios were subjected to electrophoresis for 15 min on a 1% agarose gel at 120 V using tris‐acetate (TAE) running buffer. Subsequently, the gel was visualized using UV light in a bioimaging system (Tanon Bio‐Imaging, China).

### Preparation of SN38‐NP

Copolymer mPEG‐PLys(Pep‐N3)‐PAsp(DBA) (10 mg) and 1.2 mg of SN38 were dissolved in 2 mL of dimethyl sulfoxide (DMSO). The resulting solution was then added into PBS (pH 7.4) while applying sonication (using a 60 Sonic Dismembrator from Fisher Scientific). Subsequently, the solution was dialyzed (MWCO: 14 kDa) against PBS (pH 7.4) for 24 h. After dialysis, the solution was concentrated via ultrafiltration and filtered through a 220 nm syringe filter to obtain SN38‐NP. The obtained SN38‐NP was stored at 4 °C for further experimental use.

### Preparation of si/SN38‐NP

The si‐NP solution and SN38‐NP solution were mixed in a certain ratio and incubated at 25 °C for 2 h to obtain si/SN38‐NP through DBCO‐azide click reaction. The optimal mixture ratio of si‐NP and SN38‐NP was determined by fluorescence resonance energy transfer (FRET), where FITC‐labeled siRNA was used as the donor and Cy3‐labeled interlayer of SN38‐NP as the acceptor.

### Characterization of Polymers and Nanoparticles

^1^H NMR spectra of the polymers were acquired using a Varian Unity 400 MHz Spectrometer (Varian, USA). FTIR spectra were recorded on a FTIR spectrometer (Nicolet/Nexus 670, USA) using KBr pellet as the sample preparation, covering the range of 4000 to 500 cm^−1^ with a resolution of 2 cm^−1^. Molecular weight distributions of the polymers were analyzed using a gel permeation chromatography (GPC) system (Water Breeze, USA) with PEG as the calibration standard. The GPC analysis was conducted with a mobile phase of DMF containing LiBr (1 g L^−1^) at a flow rate of 1.0 mL min^−1^, maintained at 35 °C. The morphologies of the nanoparticles were observed and captured using a TEM (Hitachi H‐7650, Japan) operated at 120 kV. A 4 µL solution with a concentration of 0.2 µg µL^−1^ was deposited onto a copper grid coated with amorphous carbon and subsequently air‐dried. The copper grid was then stained with a 1 wt.% phosphotungstic acid solution. Sizes and zeta potentials of the nanoparticles were determined using a Zetasizer Nano ZS instrument (Malvern Instruments Ltd., UK) at a temperature of 25 °C.

### Determination of the Loading Content of SN38

Lyophilized si/SN38‐NP (2 mg) were dissolved in 3 ml of isopropanol (IPA). The fluorescence intensity of SN38 in IPA at 550 nm was measured using a fluorescence spectrometer (RF‐5301 PC, Shimadzu) under 380 nm excitation. The SN38 concentration was calculated using a calibration curve for SN38 in IPA. The loading content (LC) of SN38 was calculated using the following formulas:

(1) LC=(massofSN38/massofsi/SN38−NP)×100%

### Determination the Conjugated Ratio of si‐NP to SN38‐NP in si/SN38‐NP by FRET

The copolymer mPEG‐PLys(Pep‐N_3_)‐PAsp(DBA), which was labeled with Cy3 through the reaction of Cy3‐NHS and the remaining amino groups of the PLys block, was used to prepare SN38‐NP. FITC‐labeled siRNA was used to prepare si‐FITC‐NP. The fluorescence spectra of si/SN38‐NP solutions with various mass ratios of si‐FITC‐NP / SN38‐NP (0/200, 3/200, 4/200, 5/200, 6/200, and 7/200) were measured using a fluorescence spectrometer (RF‐5301 PC, Shimadzu) under 490 nm excitation.

### Assessment of the MMP2‐Sensitive Release of si‐FITC‐NP from si‐FITC/SN38‐NP by FRET

To evaluate the MMP2‐sensitive release of si‐FITC‐NP from si‐FITC/SN38‐NP by FRET, the solution of si‐FITC/SN38‐NP containing Cy3‐labeled SN38‐NP and FITC‐labeled si‐NP was incubated with 10 nm MMP2. At certain time intervals, the emission spectra of FITC and Cy3 were measured with an excitation wavelength of 490 nm.

### In Vitro SN38 Release

To investigate the release of SN38 from si/SN38‐NP in various pH (7.4, 6.8, 5.5), 2 mL of si/SN38‐NP solution was filled into a dialysis bag (MWCO:14 kDa) which was immersed in centrifuge tubes (8 mL of PBS containing 0.5% Tween 80 at various pH in each tube) and shaken at 37 °C. At certain time intervals, 2 mL of the solution outside the dialysis bag was replaced with the same volume of fresh buffer solution. The removed solutions were lyophilized and subsequently redissolved in isopropanol for fluorescence measurement. The fluorescence intensity at 550 nm under excitation at 380 nm was used to calibrate the SN38 concentration. The cumulative amount of released SN38 was calculated using pre‐established calibration curve.

### Cellular Uptake

MC38 cells were seeded on 24‐well coverslips at a density of 2 × 10^4^ cells per well and cultured for 24 h. After staining with DAPI, cells were incubated with si‐NP or si/NR‐NP for either 2 or 4 h, followed by CLSM observation (Zeiss LS980).

The cellular uptake levels of si‐NP and si/NR‐NP were monitored by FCM analysis. MC38 cells were seeded on 6‐well plates (5 × 10^4^ cells per well) and cultured for 24 h. Then, cells were incubated with si‐NP or si/NR‐NP for 4 h. After washing with PBS twice, cells were subjected to FCM analysis (Beckman).

### RNase Protection Assay

To examine the resistance of si‐NP against nuclease‐mediated degradation, 0.5 µg siRNA and the si‐NP were incubated with RNase (50 U mL^−1^) at 37 °C, respectively. One hour later, digestion was terminated by adding RNase inhibitors (2 U µL^−1^). Then the NPs were disrupted by heparin sodium (2 U µL^−1^) and DTT (8 mm). The integrity of siRNA was examined by electrophoresis. Free siRNA was used as a control.

### Lysosome Escape

MC38 cells were seeded on 24‐well coverslips at a density of 2 × 10^4^ cells per well and cultured for 24 h. Cells were then incubated with si/B‐NP loaded with FAM‐siRNA for 4 or 24 h. After washing with PBS for twice, cells were stained with Lyso‐tracker Red (diluted 1:20 000, 30 min) and DAPI followed by CLSM observation. The co‐localization ratio between FAM‐siRNA and Lyso‐tracker Red stained lysosomes was calculated by Image J software.

### Transfection Efficiency

Cells were seeded in wells of 24‐well coverslips at a density of 2 × 10^4^ cells per well and incubated overnight, and then treated with si/B‐NP (siRNA = 50 or 100 nm). After a 4 h incubation, the cells were washed and stained with DAPI. The transfection efficiency was observed by CSLM (Zeiss LS980).

### In Vitro Gene‐Silencing Efficiency

Cells were seeded in wells of 6‐well plates at a density of 5 × 10^4^ cells per well and incubated overnight, and then treated with or without si/B‐NP (siRNA = 100 nm). After a 4 h incubation, the cells were cultured in fresh medium and allowed to grow. The knockdown efficiency of DDR1 was detected by qPCR and WB at 24 and 48 h after transfection, respectively.

### Real‐Time qPCR

Total RNA was extracted from cell culture using FastPure Cell/Tissue Total RNA Isolation Kit V2. Reverse transcription was performed using 1 µg of total RNA with HiScript III All‐in‐one RT SuperMix perfect for Qpcr. Quantitative real time PCR was performed using the QuantStudio 5 Real‐Time PCR System (Applied Biosystems) with Taq Pro Universal SYBR qPCR Master Mix from 50 ng of the reverse transcription in a final volume of 10 µL and a hybridization temperature of 60 °C. The primer sequences are shown in Table S3 (Supporting Information). The threshold cycle (Ct) value for each gene was normalized to the Ct value for β‐actin relative level of expression.

### Patient‐Derived Organoids

Organoid culture was performed as reported previously.^[^ ^44^ ^]^ For the drug sensitivity test, organoid viability was assessed with an Organoid Viability ATP Assay Kit, following the manufacturer's recommendations. Briefly, Organoids were digested into single cells, suspended in Matrigel, seeded at a density of 10000 cells per well in 96‐well black plate. After 24 h, organoids were exposed to various treatments indicated in the legend (CPT‐11: 20 µm, SN38: 6 µg mL^−1^, siRNA‐Ddr1: 1 µg mL^−1^) for 72 h at 37 °C. At the end of the incubation, the test reagents were added, and the chemiluminescence values were measured after 20 min at RT by i3x Microplate Reader (SpectraMax).

For the penetration experiment. Organoids were suspended in Matrigel and seeded on glass‐bottomed dishes (20 mm in diameter, and culture until organoids were more than 100 µm in diameter. Then, the organoids were exposed to different treatments as indicated for 30 min at 37 °C. Nucleus were stained with Hoechst 33 342 (10 µg mL^−1^, 5 min) followed by CLSM z‐stack scanning.

### CCK‐8 Assay

Cell viability was tested with CCK‐8 Assay Kit, as recommended by the manufacturer. Briefly, cells were seeded on CAFs‐derived ECM at a density of 20000 cells per well in 24‐well plate. After 24 h, and then cultured in a complete medium with various inhibitors for a further 48 h, as shown in the diagram. At the end of the incubation, add the mixture of CCK‐8 solution and DEME medium (1:10) to the substrate and incubation for 1 h at 37 °C. Then the absorbance value was measured using Microplate Reader (BioTek) at 450 nm. All tests were conducted in triplicate and data normalized to untreated controls (set at 100% viability).

### Live Imaging

For in vivo biological distribution study, the co‐injection C57BL/6 tumor‐bearing mice (≈500 mm^3^) were i.v. injected with either si‐NP or si/B‐NP (Cy7‐siRNA: 1 mg kg^−1^). At predetermined time intervals, the mice were imaged using an In Vivo Imaging System. In a parallel study, mice were sacrificed 48 h after injection. The major organs (heart, liver, spleen, lung, and kidney) and tumor tissues were harvested and imaged (λex = 633 nm, λem = 678 nm).

### In Vivo Anti‐Tumor Immunotherapy efficacy

The co‐injection C57BL/6 tumor‐bearing mice model was construct. After 8 days, the mice were randomly divided into six groups, with each group having an average tumor volume of 100 mm^3^ (*n* = 6). Subsequently, PBS, CPT‐11, SCR/SN38‐NP, si/B‐NP, si/In‐SN38‐NP and si/SN38‐NP were administered intravenously in the mice (CPT‐11: 20 mg kg^−1^, SN38: 6 mg kg^−1^; siRNA‐Ddr1: 1 mg kg^−1^). Tumor progression in mice was monitored through the tumor volume and body weight every 3 days. After treatment end point, the mice were sacrificed, and the tumor tissues was collected for analysis.

### ELISA

Mouse IFN‐gamma ELISA Kit, Mouse IL‐6 ELISA Kit, Mouse TNF‐alpha ELISA Kit, Mouse IL‐2 ELISA Kit, and Mouse Gzms‐B ELISA Kit were used, following the provided kit instructions. Simply, 100 µL of standard, sample and blank were added to their retrospective wells, Cover the plate with a membrane seal and incubate at room temperature with agitation at 200 rpm for 2 h. Wash the plate five times. Hundred microliters of 1×working solution for detecting antibody was added to each well and incubated at 200 rpm for 1 h. Wash the plate again. Hundred microliters of 1×SA‐HRP working liquid was added to each well. Oscillate at room temperature 100–300 rpm and incubate for 30 min. Following another wash, 90 µL of TMB color developing liquid was added to each well, and the color was allowed to develop away from light at RT. Finally, 50 µL of a termination solution was added to each well to stop the reaction, and the absorbance was immediately measured at a wavelength of 450 nm.

### Serum Stability

The particle size of si/SN38‐NP in PBS (pH 7.4) containing 10% FBS at different time points were measured by DLS (mean ± SD, *n* = 3).

### Hemolysis

The 4% red blood cell suspension was prepared. H_2_O (positive), PBS (negative), CPT‐11, SCR/SN38‐NP, si/B‐NP, si/In‐SN38‐NP and si/SN38‐NP were added into the red blood cell suspension (CPT‐11: 0.4 mg, SN38: 0.12 mg, siRNA‐Ddr1: 20 µg). Then the mixtures were incubated in 37 °C warm bath for 2 h. After centrifugation at 3000 rpm for 3 min, removing 100 µL supernatant to 96‐well plate, the absorbance value was read at 540 nm. The hemolysis rate was calculated according to the formula:

(2) Hemolysis(%)=(Asample−Anegative)/(Apositive−Anegative)

### Statistical Analysis

Data were represented as mean ± SD, and SPSS 26.0 software was used to analyze the experimental data. Experimental biological repeats and the statistical methods used were indicated in the corresponding figure legends. The significance level α = 0.05, and *P* < 0.05 indicates statistical significance.

## Conflict of Interest

The authors declare no conflict of interest.

## Supporting information

Supporting Information

## Data Availability

The data that support the findings of this study are available from the corresponding author upon reasonable request.
