# Supplementary material for: Overcoming the Tumor Collagen Barriers: A Multistage Drug Delivery Strategy for DDR1‐Mediated Resistant Colorectal Cancer Therapy
Source: Adv Sci (Weinh). 2024 Jul 2;11(33):2402107. doi: 10.1002/advs.202402107 (PMC11434232; doi:10.1002/advs.202402107)
Supplement: Supplementary file 1 — Supporting Information [file ADVS-11-2402107-s001.docx]

**Supporting information**

**Table S1. Clinical characteristics of patients in the cohort**

| **Variable** | **DFS≥5 years** | **DFS＜5 years** |
| --- | --- | --- |
| **Sex, No. (%)** |  | |
| Male | 26 (54.2) | 26 (56.5) |
| Female | 22 (45.8) | 20 (43.5) |
| **Age, median (IQR), years** | 56 (47-65) | 59.5 (53-70) |
| **Location, No. (%)** |  | |
| Colon | 16 (33.3) | 28 (60.9) |
| Rectum | 32 (66.7) | 18 (39.1) |
| **Histology type, No. (%)** |  | |
| Adenocarcinoma | 44 (91.7) | 40 (87.0) |
| Mucinous adenocarcinoma | 4 (8.3) | 5 (10.9) |
| Papillary adenocarcinoma | 0 (0.0) | 0 (0.0) |
| Signet ring cell | 0 (0.0) | 1 (2.1) |
| Squamous | 0 (0.0) | 0 (0.0) |
| Unknown or not stated | 0 (0.0) | 0 (0.0) |
| **Differentiation, No. (%)** |  | |
| Well (G 1) | 19 (39.6) | 17 (37.0) |
| Moderate (G 2) | 22 (45.8) | 21 (45.6) |
| Low (G 3) | 5(10.4) | 3 (6.5) |
| Unknown or not stated | 2 (4.2) | 5 (10.9) |
| **AJCC stage, No. (%)** |  | |
| Ⅱ | 36 (75.0)  ⅡA 22 (45.8)  ⅡB 14 (29.2) | 24 (52.2)  ⅡA 13 (28.3)  ⅡB 11 (23.9) |
| Ⅲ | 12 (25.0)  ⅢA 4 (8.3)  ⅢB 5 (10.4)  ⅢC 3 (6.3) | 22 (47.8)  ⅢA 2 (4.3)  ⅢB 12 (26.1)  ⅢC 8 (17.4) |
|  |  |  |
| **Clinical T stage, No. (%)** |  | |
| cT2 | 4 (8.3) | 2 (4.3) |
| cT3 | 27 (56.3) | 20 (43.5) |
| cT4 | 17 (35.4) | 24 (52.2) |
| **Clinical N stage, No. (%)** |  | |
| cN0 | 36 (75.0) | 24 (52.2) |
| cN1 | 9 (18.8) | 14 (30.4) |
| cN2 | 3 (6.2) | 8 (17.4) |

**Abbreviation:** No. number, IQR interquartile range, AJCC American Joint Committee on Cancer

**Table S2. The list of collagen features**

| NO. | Feature descriptions |
| --- | --- |
| Morphological features |  |
| 1 | Collagen area |
| 2 | Number of collagen fibers |
| 3 | Collagen fiber length |
| 4 | Collagen fiber width |
| 5 | Collagen fiber straightness |
| 6 | Collagen crosslink density |
| 7 | Collagen crosslink space |
| 8 | Collagen orientation |
| Intensity features |  |
| 9-14 | Histogram based-Mean, Variance, Skewness, Kurtosis, Energy and Entropy |
| Textural features |  |
| 15-94 | Contrast, correlation, energy and homogeneity from the GLCM given five different pixel distances with four different directions |
| 95-142 | Mean and variance in the convolution over the image with the Gabor filter at four scales with six orientations |

**Abbreviation:** GLCM, gray level co-occurrence matrix


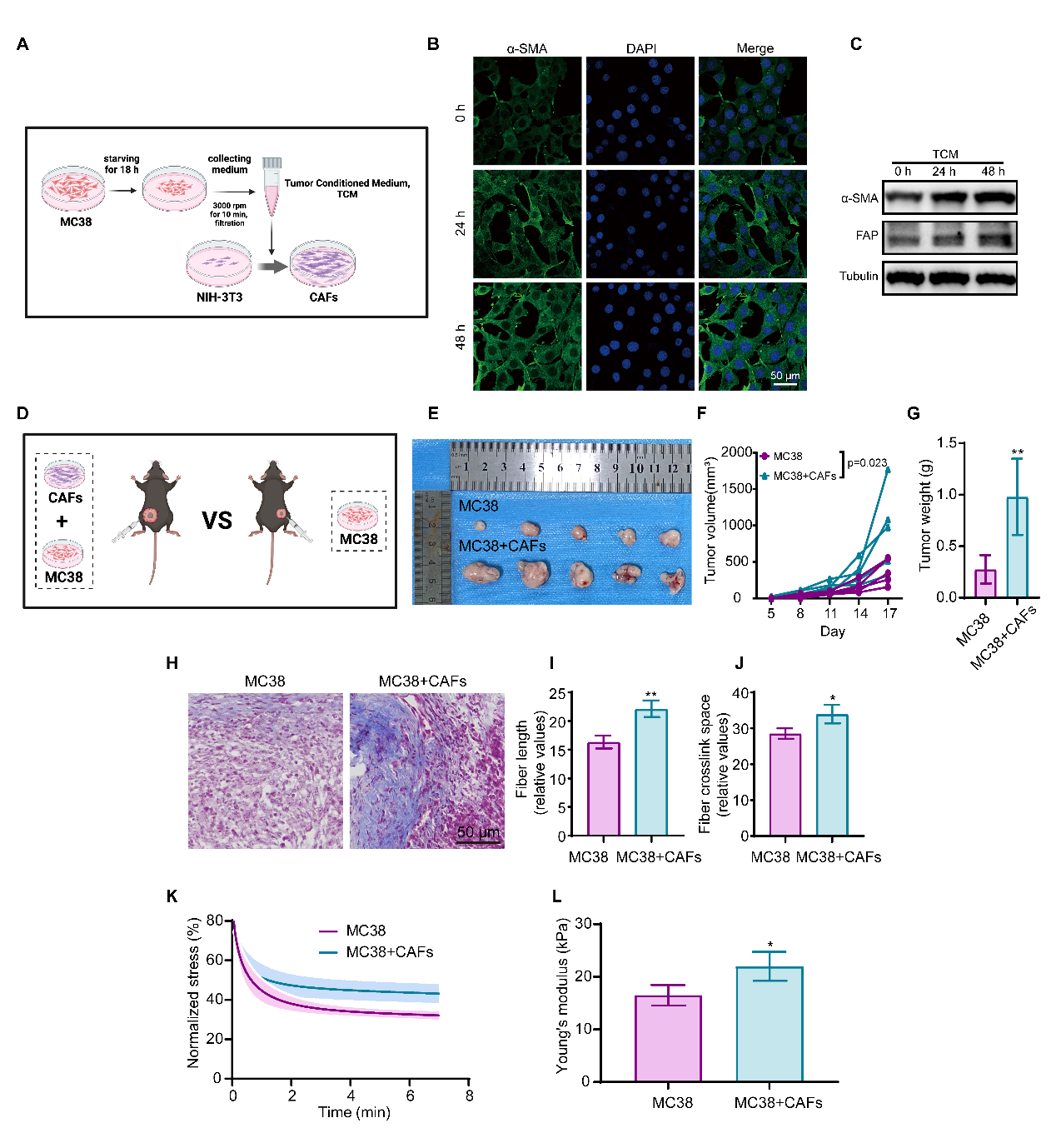


**Figure S1. The co-injection of fibroblasts and tumor cells promoted collagen deposition.**

**A.** Schematic diagram of the activation of CAFs (Created with BioRender.com).

**B.** Immunofluorescence images of anti-α SMA antibody staining indicated for the activation of NIH-3T3 cells into CAFs. Scale bar, 50 µm.

**C.** Immunoblotting of protein extracts from NIH-3T3 cells stimulated for indicated time with tumor conditioned media (TCM) collected from MC38 cells.

**D.** Schematic diagram of the establishment of CAFs-mediated resistant mice model through subcutaneous co-injecting CAFs and MC38 cells (Created with BioRender.com).

**E.** Photographs of the tumor excised from MC38 and MC38+CAFs tumor-bearing mice on 17 d.

**F.** Individual tumor growth curves of the tumors of the two groups. n=5. MANOVA Of Repeated Measuring, two tailed.

**G.** Average tumor weight of each tumor model group. n=5. Student’s t-test, two tailed. **P*<0.05, ***P*<0.01, ****P*<0.001, *****P*<0.0001.

**H.** Representative images of Masson staining of tumor tissues. Scale bar, 50 µm.

**I-J.** The collagen features (fiber length and fiber crosslink space) extracted from Masson stanning images were quantitatively analyzed by MATLAB software. n=3. One-way ANOVA, two tailed. **P*<0.05, ***P*<0.01, ****P*<0.001, *****P*<0.0001.

**K-L.** Stress relaxation of tumors in the two group. Shaded regions are s.d. of normalized stresses of different samples. Young’s modulus of as-treated tumors. n=3. Student’s t-test, two tailed. **P*<0.05, ***P*<0.01, ****P*<0.001, *****P*<0.0001.


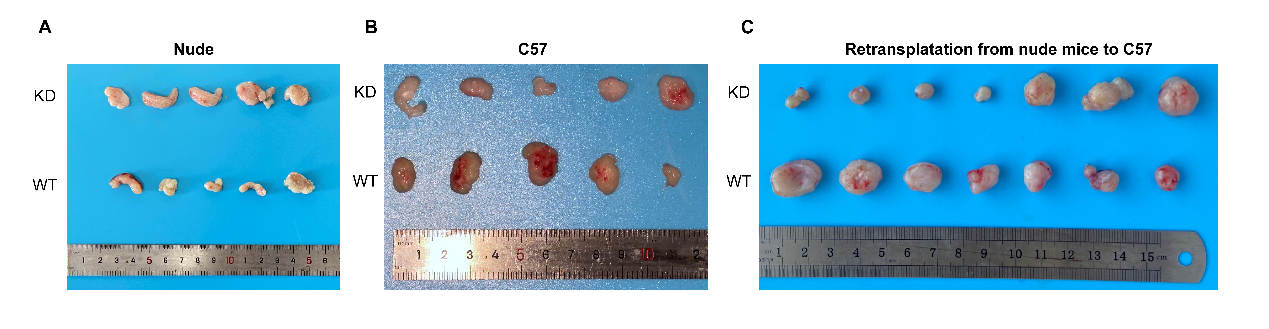


**Figure S2. Photographs of the tumors were excised from different group.**

**A.** Photographs of the tumors were excised from immunodeficient nude mice mode.

**B.** Photographs of the tumors were excised from immunocompetent C57BL/6 mice.

**C.** Photographs of the tumors were excised from mice model, where MC38 tumors were grown on immunodeficient hosts, following by cutting the tumors into approximately 60 mg pieces and transplanted into C57BL/6 mice.


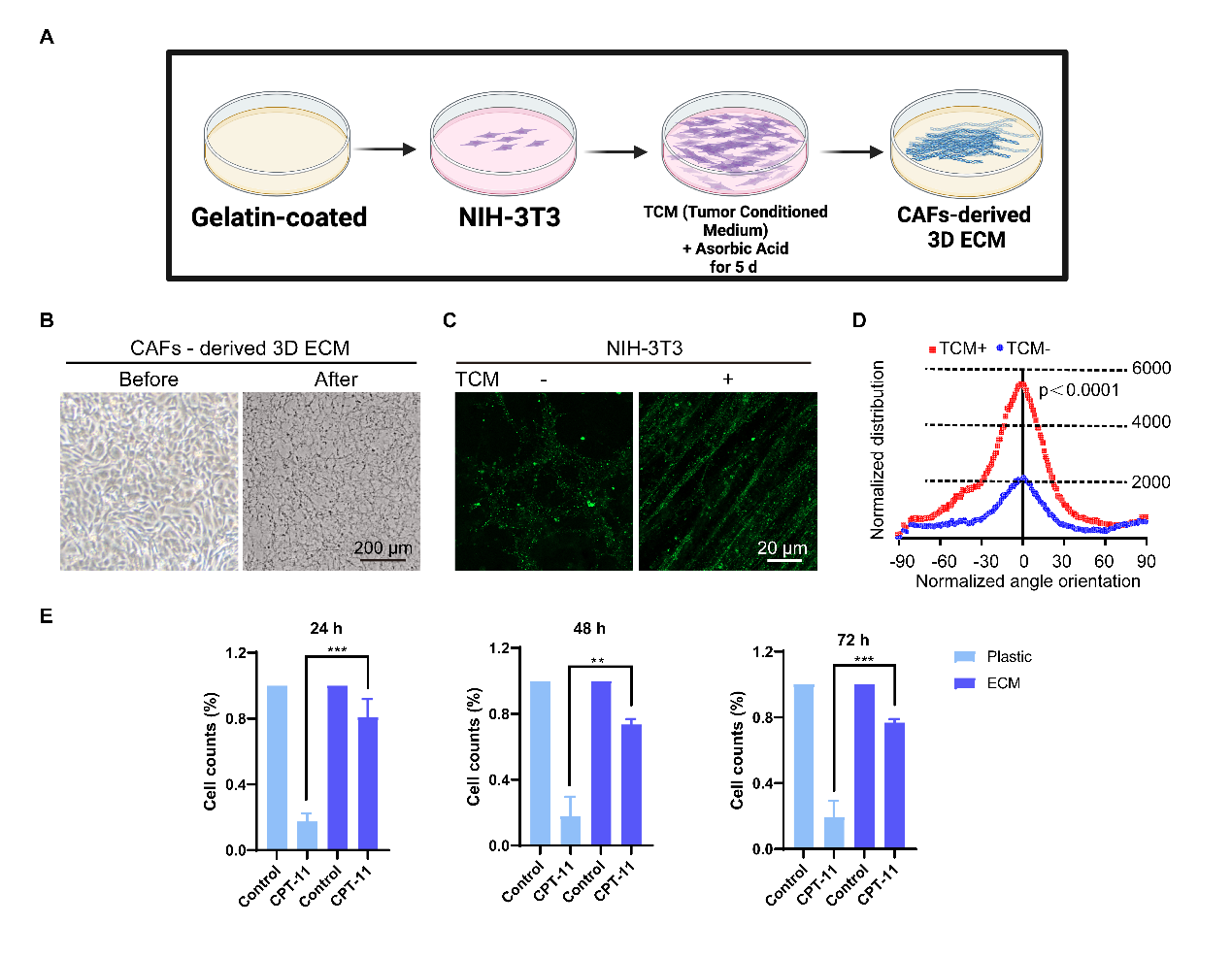


**Figure S3. The establishment of CAFs-derived 3D ECM**

**A.** Schematic diagram of the establishment of CAFs-derived ECM (Created with BioRender.com).

**B.** CAFs-derived 3D matrices before and after extraction process. Scale bar, 200 µm.

**C-D.** Immunofluorescence analysis of collagen fibers in decellularized ECM produced by NIH-3T3 cells with or without the stimuli of MC38 tumor conditional medium (TCM). Scale bar, 20 µm. The normalized distribution of collagen fiber orientation was quantified using Image. n=3. Mann-Whitney Test. two tailed.

**E.** Quantification of MC38 cell proliferation at different time points after treatment by CPT-11, which were plated on plastic dishes or the indicated CAFs-derived 3D ECM. The values in control were standardized as 1. n=3. Student’s t-test, two tailed. **P*<0.05, ***P*<0.01, ****P*<0.001, *****P*<0.0001.


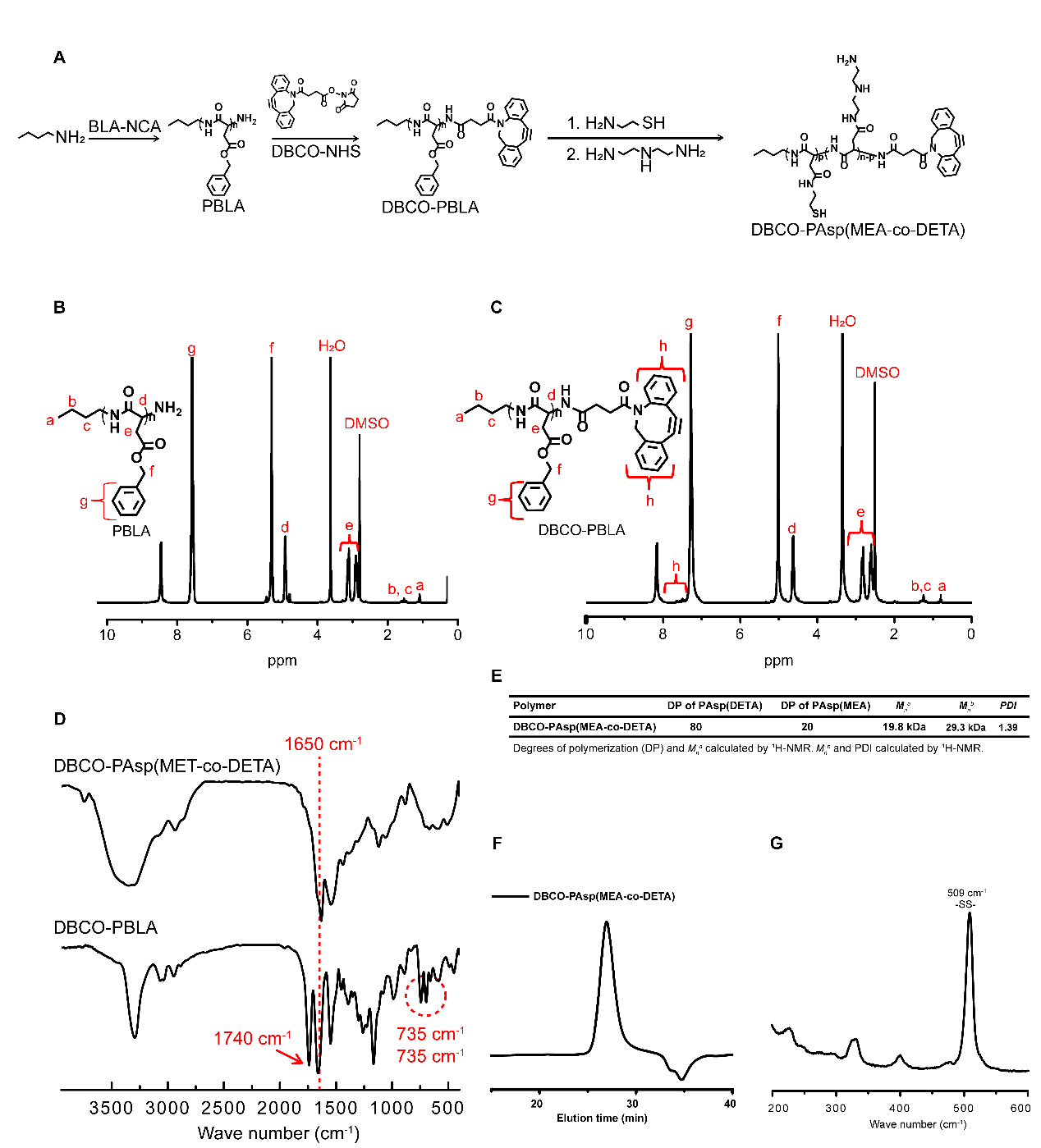


**Figure S4. Confirmation the successful synthesis of DBCO-terminated and thiol-pendant cationic polymer DBCO-PAsp(MEA-co-DETA)**

**A.** The synthetic route of cationic polymer DBCO-PAsp(MEA-co-DETA).

**B.** ^1^H-NMR spectrum of polymer PBLA in DMSO-d6.

**C.** ^1^H-NMR spectrum of polymer DBCO-PBLA in DMSO-*d*6.

**D.** FTIR spectra of DBCO-PBLA and DBCO-PAsp(MEA-co-DETA). After aminolysis reaction, the characteristic peaks at 1740 cm^-1^ of ester (s, νC=O, ester) and at 735 and 695 cm^-1^ of benzene (s, γC-H, benzene) disappeared, indicating that the aminolysis reaction was complete.

**E.** The molecular weight of the polymer DBCO-PAsp(MEA-*co*-DETA).

**F.** GPC curve of copolymer mPEG-PLys(Pep-N3)-PAsp(DBA).

**G.** Raman spectrum of si-NP. The characteristic peak at 509 cm^-1^ of disulfide bonds indicated that si-NP was crosslinked via disulfide bond.


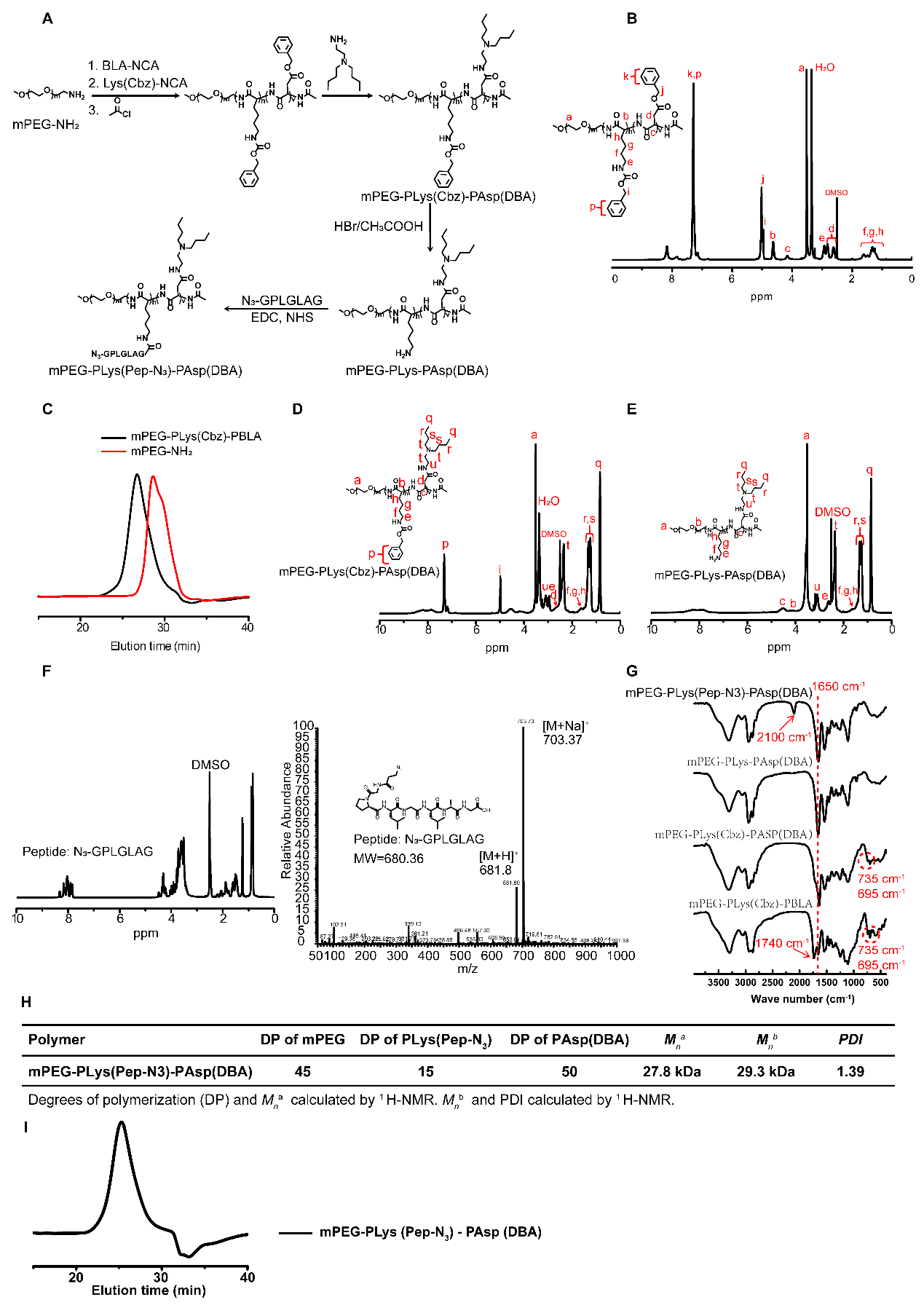


**Figure S5. Confirmation the successful synthesis of the polymer mPEG-PLys(Pep-N3)-PAsp(DBA)**

**A.** The synthetic route of MMP2-sensitive copolymer mPEG-PLys(Pep-N_3_)-PAsp(DBA) .

**B.** ^1^H-NMR spectrum of polymer mPEG-PLys(Cbz)-PBLA in DMSO-d6.

**C.** GPC curve of copolymer mPEG-PLys(Cbz)-PBLA (Mw/Mn=1.38), in DMF containing LiBr (1 g/L) at a flow rate of 1.0 mL/min. mPEG-PLys(Cbz)-PBLA showed a unimodal molecular weight distribution in chromatograms and a higher molecular weight than that of macro-initiator (mPEG-NH2).

**D.** ^1^H-NMR spectrum of polymer mPEG-PLys(Cbz)-PAsp(DBA) in DMSO-d6.

**E.** ^1^H-NMR spectrum of polymer mPEG-PLys-PAsp(DBA) in DMSO-*d*6.

**F.** ^1^H-NMR spectrum and mass spectrum of the MMP2-sensitive peptide (N_3_-GPLGLAG).

**G.** FTIR spectra of mPEG-PLys(Cbz)-PBLA, mPEG-PLys(Cbz)-PAsp(DBA), mPEG-PLys-PAsp(DBA), and mPEG-PLys(Pep-N_3_)-PAsp(DBA) . After aminolysis reaction, the characteristic peak at 1740 cm^-1^ of ester (s, *ν*_C=O_, ester) disappeared and the peaks at 735 and 695 cm^-1^ of benzene (s, *γ*_C-H_, benzene) weaken, indicating that the aminolysis reaction of PBLA block was complete. After deprotection reaction of Cbz-protected amines, the peaks at 735 and 695 cm^-1^ of benzene (s, *γ*_C-H_, benzene) disappeared. The peak at 2100 cm^-1^ was attributed to the azide absorption (*v*_azide_, N_3_), indicating that the MMP2-sensitive peptides (N_3_-GPLGLAG) were successfully grafted into the PLys block of mPEG-PLys(Pep-N_3_)-PAsp(DBA).

**H.** The molecular weight of mPEG-PLys(Pep-N_3_)-PAsp(DBA).

**I.** GPC curve of copolymer DBCO-PAsp(MEA-co-DETA).


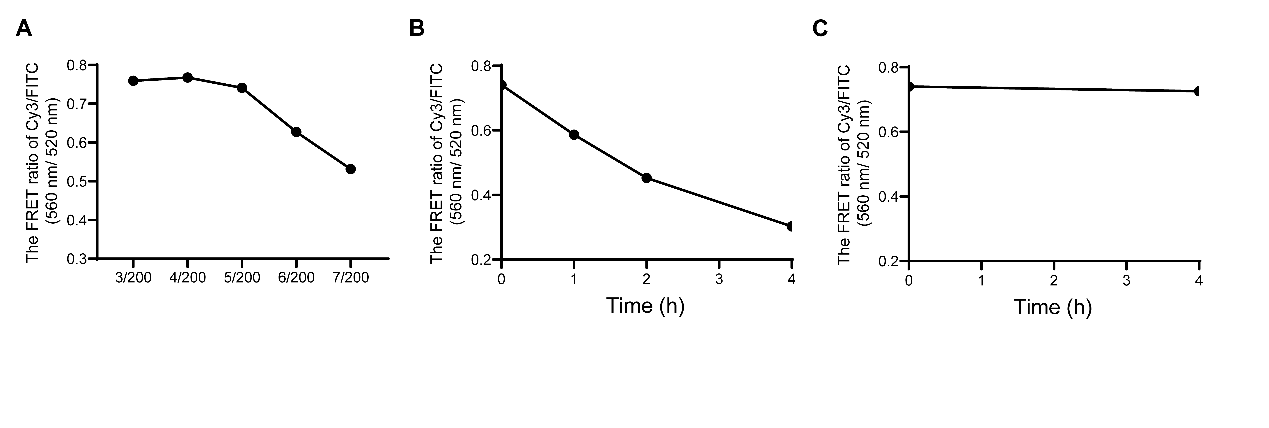


**Figure S6. The FRET ratio of Cy3/FITC**

**A.** The FRET ratio of Cy3/FITC in Figure 5J.

**B-C.** The FRET ratio of Cy3/FITC in Figure 5M. MMP2-sensitive si-FITC/SN38-NP (B), MMP2-insensitive si-FITC/In-SN38-NP (C).


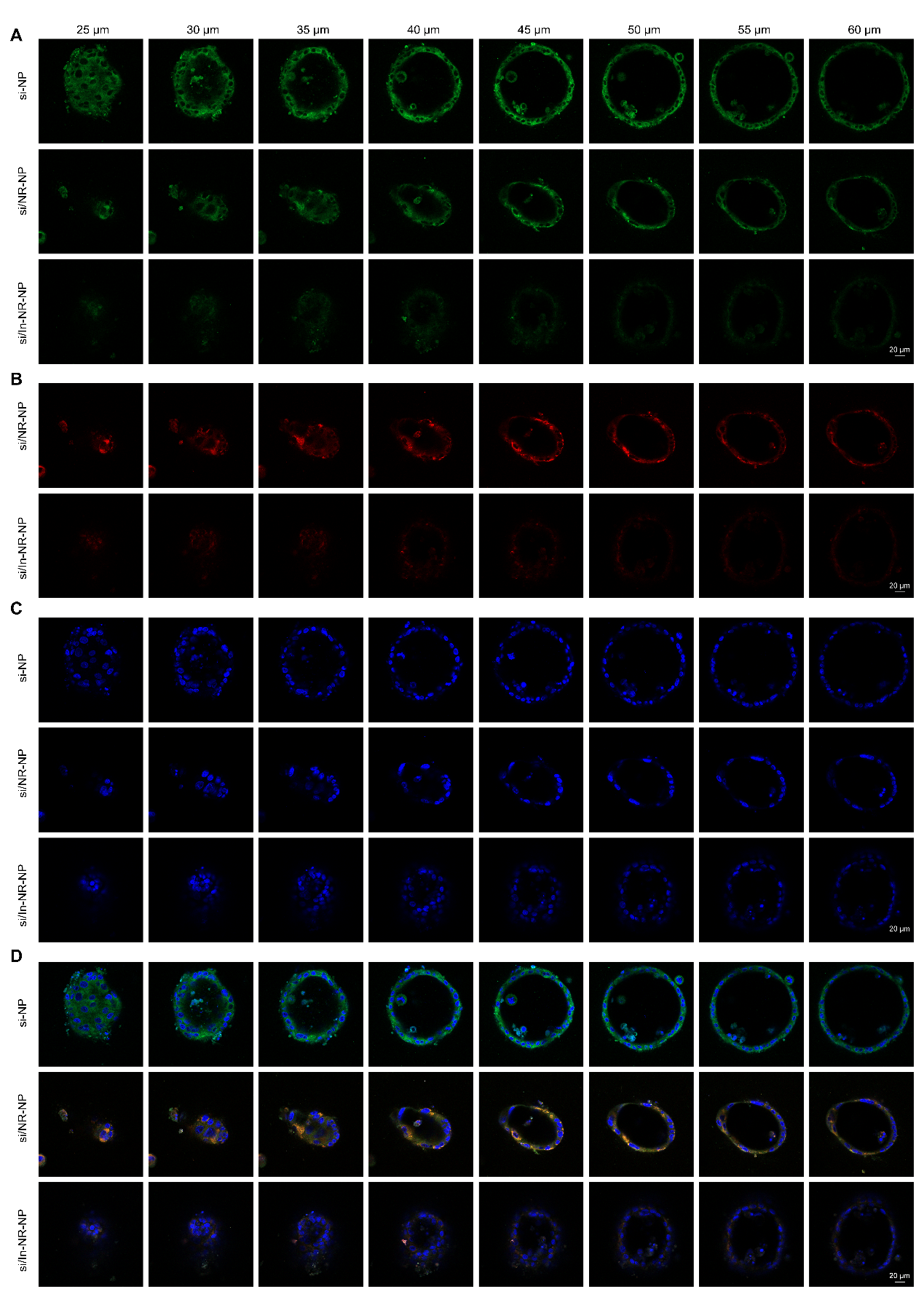


**Figure S7. The deep penetration capacity of si-NP and si/NR-NP and NR-NP**

The distribution of si-NP and si/NR-NP and NR-NP in different depth by Z-stack scan. A. FAM-siRNA (green), B. NR-NP (red), C. DAPI (blue), D. Merge. Scale bars, 20 µm.


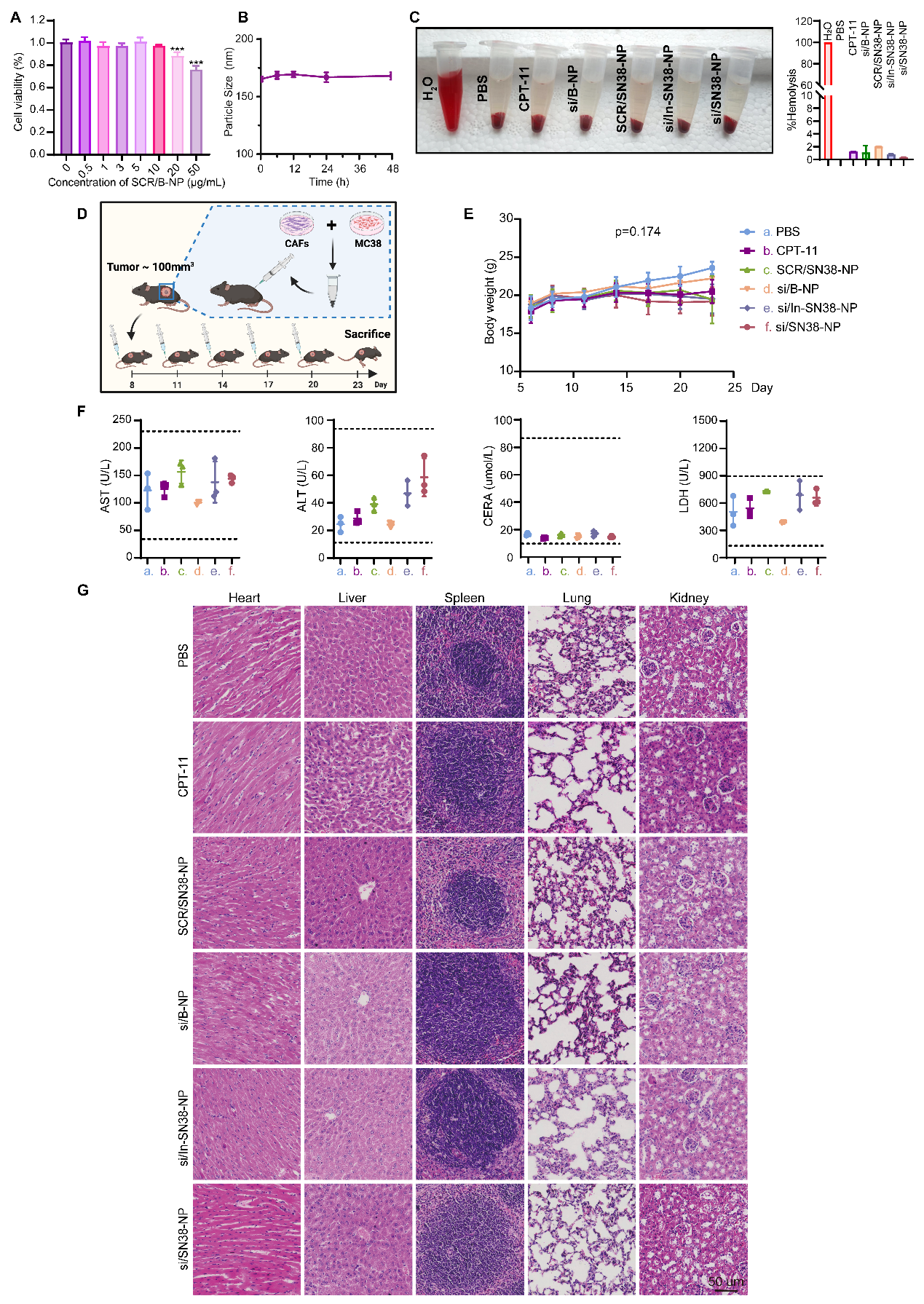


**Figure S8. The biosafety evaluation of si/SN38 NP in vitro and in vivo.**

**A.** Cell viabilities of MC38 cells incubated with SCR/B-NP at different concentrations. n=3. One-way ANOVA, two tailed. **P*<0.05, ***P*<0.01, ****P*<0.001, *****P*<0.0001.

**B.** Particle size of si/SN38-NP in PBS (pH 7.4) containing 10% fetal bovine serum (FBS) for different times measured by DLS (mean ± SD, n=3).

**C.** Hemolytic activity of si/SN38-NP. Visual inspection of the tubes containing diluted total blood after exposure to different treatments for 3 h after centrifugation. The percentages of hemolysis were shown. H_2_O and PBS were respectively used as negative control and positive control. n=3.

**D.** Schematic diagram of the timeline of C57 mice during treatment (Created with BioRender.com).

**E.** Body weight changes of C57 mice during treatment. n=6. MANOVA Of Repeated Measuring, two tailed. **P*<0.05, ***P*<0.01, ****P*<0.001, *****P*<0.0001.

**F.** Serum biochemical analysis of mice after treatment. The dashed line indicates the normal reference range of the laboratory.

**G.** H&E staining of major organs excised from mice after various treatments. Scale bar, 50 µm.
